# Process design for acidic and alcohol based deep eutectic solvent pretreatment and high pressure homogenization of palm bunches for nanocellulose production

**DOI:** 10.1038/s41598-024-57631-9

**Published:** 2024-03-30

**Authors:** Janejira Sonyeam, Ratanaporn Chaipanya, Sudarat Suksomboon, Mohd Jahir Khan, Krongkarn Amatariyakul, Agung Wibowo, Pattaraporn Posoknistakul, Boonya Charnnok, Chen Guang Liu, Navadol Laosiripojana, Chularat Sakdaronnarong

**Affiliations:** 1 Department of Chemical Engineering, Faculty of Engineering, Mahidol University, 25/25 Putthamonthon 4 Road, Salaya, Putthamonthon, Nakhon Pathom, 73170 Thailand; 2 Department of Specialized Engineering, Energy Technology Program, Faculty of Engineering, Prince of Songkla University, 15 Karnjanavanich Rd., Hat Yai, Songkhla, 90110 Thailand; 3 State Key Laboratory of Microbial Metabolism, School of Life Sciences and Biotechnology, Shanghai Jiao Tong University, 800 Dongchuan Road, Shanghai, 200240 China; 4 The Joint Graduate School of Energy and Environment, King Mongkut’s University of Technology Thonburi, 126 Pracha Uthit Road, Bang Mot, Thung Khru, Bangkok, 10140 Thailand

**Keywords:** Deep eutectic solvent, Nanocellulose, Palm empty fruit bunch, Waste utilization, Nanorods, Nanofibers, Waste management, Energy science and technology, Engineering, Nanoscience and technology, Nanoscale materials, Synthesis and processing, Two-dimensional materials, Materials science, Organic molecules in materials science, Polymers

## Abstract

This research aimed to study on nanocellulose production from palm bunch using process design and cost analysis. Choline chloride based deep eutectic solvent pretreatment was selected for high-purity cellulose separation at mild condition, followed by nano-fibrillation using mechanical treatment. Three types of choline chloride-based deep eutectic solvents employing different hydrogen-bond donors (HBDs) namely lactic acid, 1,3-butanediol and oxalic acid were studied. The optimal cellulose extraction condition was choline chloride/lactic acid (ChLa80C) pretreatment of palm empty bunch at 80 °C followed by bleaching yielding 94.96%w/w cellulose content in product. Size reduction using ultrasonication and high-pressure homogenization produced nanocellulose at 67.12%w/w based on cellulose in raw material. Different morphologies of nanocellulose were tunable in the forms of nanocrystals, nano-rods and nanofibers by using dissimilar deep eutectic solvents. This work offered a sustainable and environmentally friendly process as well as provided analysis of DES pretreatment and overview operating cost for nanocellulose production. Application of nanocellulose for the fabrication of highly functional and biodegradable material for nanomedicine, electronic, optical, and micromechanical devices is achievable in the near future.

## Introduction

Nanocellulose, as an environmentally friendly and biodegradable nanomaterial with green credentials, has garnered significant attention in the nanomaterial community over the past decade. This attention is primarily due to its non-toxicity, eco-friendliness, natural biocompatibility, and outstanding physicochemical properties, including its low density, expansive specific surface area, high crystallinity, impressive mechanical strength, and modulus^1,2^. These advantageous characteristics position nanocellulose as having substantial potential across various fields, including 3D printing materials^3^, biomedicine^4,5^, food packaging^6^, functional nanopapers^7^, pickering emulsions^8^, flexible electronics^9^, solar cells^10^, and wearable sensors^11^.

In recent years, as interest in nanocellulose has grown, numerous studies have explored its preparation methods, encompassing cellulose nanofibers (CNFs) and cellulose nanocrystals (CNCs). CNCs typically refer to small rod-like nanocrystals with widths and lengths ranging between about 5 to 20 nm and 50 to 500 nm, respectively. The dimensions of CNCs are directly tied to the source of lignocellulosic materials and the manufacturing processes used^12^. CNFs, on the other hand, are generally described as slender nanofibers with diameters between 10 and 100 nm and lengths extending to several micrometers^13^.

It was reported that CNCs were firstly produced from bleached sulfite pulp using 2.5 N sulfuric acid^14^. This breakthrough laid the groundwork for subsequent methods of producing CNCs through mineral acid hydrolysis, including the use of acids like H_2_SO_4_, HCl, and H_3_PO_4_^15–17^. Later, the initial extraction of CNF from wood was accomplished through a high-pressure homogenization process at 55 MPa^18^. This marked the beginning of exploration into various mechanical techniques for CNF extraction, such as ultrasonication^19^, grinding^20^, microfluidization^21^, ball milling^22^, and high-speed blending^23^. However, it is important to note that mechanical treatments alone cannot completely break the robust hydrogen bonds between cellulose chains, making it challenging to isolate individual fibrils^24^. Even when nanofibrils are successfully separated, relying solely on mechanical processing is hindered by its high energy consumption and limited product consistency^25^. To address these challenges, a viable approach is to introduce appropriate biological or chemical pretreatments before mechanical operations to yield nanocellulose. These pretreatment methods encompass technologies like TEMPO oxidation^26^, carboxymethylation^27^, phosphorylation^28^, enzyme hydrolysis^29^, and more^30^. However, despite their effectiveness in producing nanocellulose, the industrialization of these conventional processes faces significant obstacles related to environmental impact and economic feasibility.

Lately, ionic liquid (IL) pretreatment has emerged as a promising solvent for nanocellulose production, primarily due to its non-flammable nature, high thermal stability, and low vapor pressure^31^. However, the drawbacks of IL, including its toxicity and high cost, limit its practical use in nanocellulose fabrication. In contrast, deep eutectic solvent (DES), which shares similar characteristics with IL but is more affordable and less toxic, is gaining traction as a viable option for nanocellulose preparation^32^. Typically, DES is categorized as an eco-friendly solvent composed of a hydrogen bond acceptor (HBA) and a hydrogen bond donor (HBD) at specific temperature conditions^33,34^. The attractive forces like van der Waals interactions and hydrogen bonding between the HBA and HBD components in DES result in a lower melting point for the formed transparent DES compared to the individual constituents^35,36^. Moreover, DES offers several advantages, including ease of synthesis, customizable structure, environmental friendliness, recoverability, and so forth^37,38^. Additionally, a majority of the constituents used in DES systems are renewable and biodegradable, aligning well with the principles of green chemistry^37^. In recent times, these benefits have led to the widespread adoption of DES for pretreating various biomass feedstocks to dissolve lignocellulose and extract nanocellulose^39–41^.

Previous research has shown that acidic DESs, such as those based on choline chloride (ChCl) combined with acetic acid, formic acid, citric acid, or lactic acid, exhibit a high solubility for lignin. This, in turn, has a substantial positive impact on enhancing sugar yields^42^. An effective removal of lignin, and separation of cellulose was successfully reported from moso bamboo using a combination of ChCl and lactic acid at moderate temperature under 100 °C^43^. On the other hand, certain alkaline or neutral DESs, such as those composed of ChCl with urea, glycerin, or imidazole, have demonstrated limited effectiveness in removing lignin and have a less pronounced impact on saccharification^44^. In light of these findings, acidic DES treatments have gained prominence as a green, environmentally friendly, and sustainable approach for nanocellulose extraction in recent years. Among the various acidic DES options, those based on carboxylic acids and ChCl are recognized as an optimal method for cellulose hydrolysis and the production of nanocellulose. In addition, three types of primary alcohols or butanediols, each with different functional group positions (1,2-butanediol, 1,3-butanediol, and 1,4-butanediol), have been purposely designed and synthesized as HBDs for DESs. These butanediols are combined with ChCl serving as the HBA and resulted in a unique electrostatic property of each butanediol investigated that could influence the solubility of solute to be extracted^45^. This combination of ChCl and butanediols has been successfully employed in the extraction process for bioactive compounds or drugs^46,47^. Despite the promising benefits of DESs as green solvents, there are significant challenges when it comes to their extensive use in nanocellulose production. The most apparent issues are their high viscosity and high cost, which result in a low mass concentration of dissolution and slow solvent-transfer operations. These challenges need to be addressed to fully leverage the potential of DESs in large-scale nanocellulose production^45^.

To the best of our knowledge, there have been no specific studies that have compared acidic and alcohol-based DESs for refining lignocellulosic biomass and achieving massive nanocellulose production. Consequently, various types of acidic based DESs (ChCl/oxalic acid and ChCl/lactic acid) and alcohol based DESs (ChCl/1,3-butanediol) were investigated for cellulose extraction from palm empty fruit bunch at varied reaction temperatures. Cellulose nano-fibrillation using ultrasonication and subsequent high-pressure homogenization was applied and the unique morphology of nanocellulose products from different DESs was reported.

## Experimental

In this research, analytical grade chemicals for DESs synthesis namely choline chloride, lactic acid, 1,3-butanediol and oxalic acid were purchased from Alfa Aesar (Thermo Fisher Scientific). EFB is one of the oilseed crop residues found abundantly in Southeast Asia^48^. It was supplied from a Palm Oil Mill, Thailand. Commercial cellulose, sodium chlorite, and acetic acid were acquired from Sigma-Aldrich.

### Chemical engineering process design for nanocellulose production

Prior to experimental work on nanocellulose production from palm empty fruit bunch, the literature survey was performed in two sections: (1) lignocellulosic biomass pretreatment to obtain high cellulose yield and (2) nanocellulose production from pretreated cellulose which was moreover separated into chemically acid hydrolysis process and physical/mechanical size reduction of cellulose to nanocellulose. The most feasible process among all literatures that could produce high yield of nanocellulose based on the same amount of raw material (palm empty fruit bunch) was selected, and the experiments on EFB pretreatment, high-purity cellulose making and nanocellulose production were carried out.

### DESs preparation and EFB pretreatment

Preparation of DESs was conducted at different molar ratios of HBA (ChCl) and three HBDs including lactic acid, 1,3-butanediol, and oxalic acid at 1:10, 1:2 and 1:1, respectively. The two chemicals were mixed at room temperature, heated to 80 °C under vigorous stirring for 1 h to get clear solution, and cooled down to room temperature (30 °C) for use. Three types of DESs were named as ChLa, ChBu and ChOx, respectively.

For EFB delignification pretreatment in DESs, EFBs (− 200/+ 325 mesh) were pretreated in freshly prepared ChCl based DESs with a solid to liquid ratio of 1:10 by weight. The reaction took place at 60, 80 and 100 °C for 8 h under refluxin a round bottom flask equipped with a condenser. The slurry of EFB after DES extraction was diluted three folds with deionized water by weight, and centrifuged at 10,000 rpm for 15 min. Solid fraction was dried in an oven at 60 °C overnight for further experiment and analysis. Liquid fraction was analyzed by High performance liquid chromatography (HPLC) for the products from the chemical reaction in different DESs.

### Pulp bleaching

Solid fraction from DESs treatment was bleached to obtain more whiteness and higher purity of cellulose. Solid after DESs pretreatment was mixed with 1.5 wt% NaClO_2_ solution at a solid to liquid ratio of 1:50. The bleaching reaction was conducted at 70 °C, with stirring speed of 250 rpm for 30 min. Acetic acid was added every 30 min until pH of the mixture reached 4, and the mixture was continuously heated until 2 h^49,50^. After bleaching, the mixture was filtrated and washed with deionized water. After dried at 60 °C for overnight, photograph was taken and the whiteness (ΔE) of the bleached pulp was calculated according to Eq. (1) using Adobe Photoshop Program when the color value was expressed by CIE model^51^, which reported as L*, a*, b* color coordinates from which L* value indicates lightness, a* value is the color ranged from green to red, and b* value is the color ranged from blue to yellow. The bleaching process was carried on repeatedly until the whiteness index, ΔE, reached a constant value.

1 $$\Delta E = \sqrt{{L}^{*2}+{a}^{*2}+{b}^{*2}}$$

### Nano-defibrillation using ultrasonication and high-pressure homogenization

After bleaching process, deionized water was added to bleached cellulose at the solid to liquid ratio of 1:100. The cellulose-water mixture was fibrillated to obtain fine and small fiber using ultrasonic generator at 20 kHz for 20 min at 40% amplitude of highest intensity ultrasonic processor (VCX 750, CT, USA) with the probe tip diameter of 13 mm for 300 mL of cellulose solution. The sample temperature during the sonication was maintained at 30 °C using an ice bath. After ultrasonication, micro-/nano-cellulose fiber suspension was then defibrillated by high-pressure homogenizer (HPH) (Microfluidics, M-110P, USA) at a constant pressure of 150 MPa and temperature of 30 °C when the total cellulose solution was used at least 300 mL for each run.

### Characterization and data analysis

Chemical composition analysis of pretreated EFB and nanocellulose from different steps was performed according to Goering and Soest Method^52^ and the detail of analysis was reported elsewhere^53^. Enzymatic digestibility of DESs pretreated sample was analyzed using a commercial cellulase (Cellic^®^Ctec2, Novozyme). Dry EFB sample (0.2 g) was mixed with 19.2 mL of 0.050 mol L^−1^ sodium acetate buffer, pH 4.80 and 0.4 mL Ctec2 enzyme (200 FPU g^−1^ dry sample)^53^. The hydrolysis took place in an incubator shaker at 50 °C for 72 h at 150 rpm, and the supernatant was analyzed by HPLC. The cellulose digestibility was calculated from the amount of glucose released from cellulose hydrolysis as shown in the Eq. (2).

2 $$Cellulose\,\, digestibility (\%) =\frac{ Amount\,\, of\,\, glucose\,\, from\,\, cellulose\,\, hydrolysis\,\, (g) }{Amount\,\, of\,\, cellulose\,\, in\,\, sample (g)}\times 100$$

Sugars (e.g., glucose, xylose, cellobiose) and organic acids (e.g., acetic acid) in liquid fraction from DES pretreatment of EFB were quantified by HPLC (Waters model Alliance 2690, USA) equipped with Aminex HPX-87H Column (7.8 mm × 300 mm, BioRad, USA) and reflective index (RI) detector. The column temperature was set at 60 °C and the 0.005 mol L^−1^ sulfuric acid was used as a mobile phase at a flow rate of 0.6 mL min^−1^^53–55^.

Fourier transform infrared spectroscopy (FT-IR) was carried out at wavenumber of 4000–400 cm^−1^ with 100 numbers of scan (Model Nicolet iS50 FT-IR, Thermo Scientific, USA). The crystal structure was measured using X-ray diffractometer (XRD) from 5° to 80° (2 theta) (Model D8 Discover, Bruker, Germany). Crystallinity index (CrI) was calculated from the difference of XRD intensity of I_200_ (cellulose crystalline structure at 22.5°) and I_Am_ (amorphous structure at 18°) according to a previous report^53^ and by Segal method^56^ (Eq. 3).

3 $$CrI \left(\%\right)=\frac{{I}_{200}-{I}_{Am}}{{I}_{200}}\times 100$$

Field emission transmission electron microscope (FE-TEM) with an accelerating voltage of 300 kV (FE-TEM, Model JEM-3100F, JEOL, Japan) was used to analyze morphology and width of nanocellulose. Viscosity of nanocellulose at 12-pass HPH were analyzed using a rheometer (Malvern model Kinexus Pro, UK) fitted with a PU20 probe and operating at 25 °C when the frequency was 1 Hz. Aspen Plus software was used for simulation of mass balance in the process and economic analysis.

## Results and discussion

### Engineering process design of nanocellulose production from lignocellulosic biomass

From the literature review, the pretreatment processes were firstly evaluated for their feasibility in terms of cellulose yield, extracted from lignocellulosic biomass (Cellulose Separation; CellSep process) using DESs as well as wastewater disposal without any recycles and economic considerations using economic assessment in Aspen software. Subsequently, chemical reaction (Chemical Process; ChemCN process) for cellulose hydrolysis to obtain nanocellulose was reviewed and compared with physical/mechanical nanofibrillation to produce nanocellulose (Mechanical Process; MechCN process).

#### Process analysis for cellulose separation (CellSep) from lignocellulosic biomass

To achieve cellulose separation from lignocellulose, an effective pretreatment process to remove lignin and hemicellulose with minimum damage of cellulose structure is required. DESs have emerged as a promising class of environmentally friendly solvents that align perfectly with the twelve principles of green chemistry^57^ demonstrating efficient performance in biomass pretreatment, and surpassing the capabilities of certain conventional solvents^58^.

Firstly, the study on enhanced disruption of EFB by ChCl deep eutectic solvents for cellulose production from our group was introduced (CellSep-A1 process) as shown in Fig. S1^59^. The maximum cellulose yield was achieved when EFBs were pretreated with DES based on ChCl and 1,3-butanediol. The reaction occurred in a hydrothermal reactor at the reaction temperature of 200 °C and the reaction time of 20 min under the N_2_ pressure of 1 MPa. It was reported that 15.77 wt% cellulose was obtained from 100 kg EFB corresponding to 40.96%w/w cellulose yield based on cellulose in raw EFB. Kumar and his colleagues studied on techno-economic evaluation of natural DES-based biorefinery of rice straw and explored the different design process scenarios^60^. The optimal condition was carried out in a reactor with DES based on ChCl and lactic acid at 121 °C and for 20 min under the pressure of 0.1 MPa yielding 10.92 wt% cellulose from rice straw (CellSep-A2 process). The cellulose yield based on cellulose content of rice straw was 27.3%w/w. Zang et al*.* conducted a study on pretreatment of switch grass for cellulose production using a techno-economic approach. One-pot biomass fractionation and furfural production were focused^61^. As shown in Fig. S1, the pretreatment was carried out in a one-pot reactor containing ChCl, sulfuric acid and methyl isobutyl ketone (MIBK) at 170 °C for 60 min under 0.76 MPa that was the most excellent condition to separate cellulose from switch grass (CellSep-A3 process). The obtained cellulose was 13.84 wt% from 100 kg dry switch grass corresponding to 40.12%w/w cellulose yield based on cellulose in switch grass. The reason for lower cellulose yield of CellSep-A2 and CellSep-A3 compared with CellSep-A1 was mainly due to cellulose breakdown during treating with ChCl/lactic acid in which acid could catalyze the breakdown of β, 1-4 glycosidic linkage to short chain of cellulose polymer.

Considering all the analyzed processes, the greatest cellulose yield after pretreatment was from CellSep-A1 process in which EFB was pretreated in DES in a combination of ChCl and 1,3-butanediol^59^. The maximum cellulose yields attained with ChCl and 1,3-butanediol were due to the preservation of cellulose integrity. The low cellulose yield while using ChCl/lactic acid and ChCl/oxalic acid was mostly caused by cellulose degradation after treatment with acid-based DESs, where acidic solutions could possibly catalyze the breakdown of β-1,4 glycosidic linkage into shorter cellulose polymer chains. In contrast, polyol-based DESs, ChCl/1,3 butanediols possess unbound hydroxyl groups that interact with lignin's free and etherified hydroxyl groups. This interaction considerably improves lignin fractionation efficiency and cellulose accessibility during the process^62^. The systematic process flow diagram of CellSep-A1 was performed as demonstrated in Fig. S2 with the cellulose yield was 40.96%w/w. The mass balance showed that cellulose input in the stream 1 was 38.5 kg h^−1^ and the extracted cellulose in the solid A which was cellulose in stream 7 was 17.6 kg h^−1^ cellulose corresponding to 45.70%w/w cellulose yield based on cellulose in EFB (Table S1).

Comparison of economic feasibility of all three processes demonstrated in Fig. S1 was additionally performed using Aspen Plus software as shown in Table S2. The result exhibited that the report from Nosri and coworkers^59^ (CellSep-A1 process) was more feasible than others where the capital cost and the operating of the process were 1.16 million USD and 33.14 million USD per year, respectively, and this caused the highest yearly profit of 33.70 million USD per year. Therefore, net present value (NPV) and internal rate of return (IRR) can be estimated to 1.25 million USD and 47%, respectively. It would provide only 3 years of payback period to recover the original investment. In conclusion, DES based on ChCl and 1,3-butanediol was selected for cellulose extraction from EFB in experimental study in the present work.

#### Nanocellulose production from cellulose-rich (pulp) fraction (chemical process; ChemCNC)

Acid hydrolysis is commonly used in cellulose hydrolysis for cellulose size reduction^63^. To select the optimal acid hydrolysis condition for this study, the best condition from highest nanocellulose yield which was observed from the previous research was examined in detail using a simulation software.

From the first research work of Bondancia and colleagues, 65 wt% of citric acid solution was mixed with eucalyptus kraft pulp and the reaction occurred in a reactor at 45 °C, 450 rpm for 6 h (ChemCNC-B1 process in Fig. S3). From this work, 23 kg CNC and 52 kg CNF were produced from 100 kg Eucalyptus cellulose kraft pulp containing 75.60 kg cellulose which was corresponded to 30.56%w/w CNC yield and 68.78%w/w CNF yield based on cellulose in kraft pulp^64^. Guo and colleagues produced nanocellulose from softwood sulfite pulp via acid hydrolysis using 64 wt% sulfuric acid at 45 °C for 2 h (ChemCNC-B2 process)^65^. It was revealed that 71 kg CNC was produced from 74 kg cellulose in softwood sulfite pulp which was 95.95%w/w cellulose yield based on cellulose content in raw material. Lastly, Wang and colleagues hydrolyzed bleached softwood kraft pulp using 0.3 wt% sulfuric acid at 160 °C, 200 rpm for 2 h (ChemCNC-B3 process)^66^. The CNC yield was reported at 15.78 kg from 87.90 kg cellulose in bleached softwood kraft pulp which was corresponded to 17.95%w/w based on cellulose in raw material.

Among three CNC production processes analyzed, ChemCNC-B2 process using 64 wt% sulfuric acid to hydrolyze cellulosic softwood sulfite pulp was the most suitable process to produce highest CNC yield (71 wt%). The process flow diagram for simulation is demonstrated in Fig. S4. The mass balance was evaluated in details using a simulation software (Table S3) and the results showed that the cellulose feed in stream 1 of 74 kg h^−1^ could generate 71 kg h^−1^ CNC at 45 °C acid hydrolysis temperature. The other unit operations required only room temperature at 25 °C, at atmospheric pressure (0.1 MPa). Therefore, this simple process seemingly requires low cost of utility as presented in the following economic assessment section. From economic analysis shown in Table S4, although ChemCNC-B2 process required highest raw material cost from cellulose pulp and chemicals for wastewater neutralization, the process consumed lowest utility cost due to low temperature and short time of acid hydrolysis, and provided greatest revenue due to highest yield of CNC produced. Therefore, the capital cost and the operation of the process were 3.02 million USD and 866.34 million USD per year, respectively. The yearly profit was 866.48 million USD per year. Consequently, the NPV and IRR can be estimated as -1.4 million USD and 2%, respectively. Relatively lengthy payback period of 21 years could possibly be reduced by adding acid recycling process. Nevertheless, detailed analysis and evaluation results showed a massive economic and environmental disadvantage from the large amount of chemicals consumed from concentrated acid hydrolysis and neutralization of acid usage. Therefore, physical/mechanical processes for nanocellulose production from cellulose-rich material was further analyzed in the following section.

#### Nanocellulose production from cellulose-rich (pulp) fraction (mechanical process; MechCN)

Initially, Li and colleagues evaluated the experiment on nano-fibrillated cellulose from wood powder using ultrasonication (MechCN-C1) as shown in Fig. S5. After ethanol solvothermal reaction at 180 °C for 80 min to remove lignin, wood powder was subjected to alkaline-hydrogen peroxide pretreatment at 50 °C for 90 min to eliminate residual lignin and hemicellulose^67^. As a result, 20.86 kg nanocellulose was produced from 23.18 kg cellulose in pretreated material by means of ultrasonication at 20 kHz for 20 min which corresponded to 90%w/w nanocellulose yield (diameter 1–9 nm) based on cellulose content.

In MechCN-C2 process, sugarcane bagasse was pretreated by steam explosion at 250 °C for 10 min and after alkaline-hydrogen peroxide delignification at 65 °C for 45 min for 2 times, cellulose was produced for 23.57 kg from 100 kg of sugarcane bagasse. High-pressure homogenization (HPH) at 138 MPa for 12 passes was applied to produce nanocellulose. This process yielded 20.15 kg nanocellulose (85.5% nanocellulose yield based on cellulose in pretreated material) with narrower range of diameter of 3–7 nm^68^. Lastly, Gao and colleagues investigated a combined ball milling (30 min) and alkaline treatment (80 °C for 2 h) to produce nanocellulose from wheat straw (MechCN-C3 process). Microcrystalline cellulose (CMF) (~ 10 µm in diameter) was produced at 92.30%w/w CMF yield based on cellulose in raw material^69^.

The detailed process flow diagram of MechCN-C3 process that yielded highest microcrystalline cellulose was illustrated in Fig. S6. Table S5 additionally demonstrated the simulation results showing 35 kg h^−1^ cellulose in wheat straw in stream 1 was turned to CMF at 30.69 kg h^−1^. Economical parameters evaluated in Table S6 demonstrated that although MechCN-C3 process provided highest CMF yield and gave a short payback period (3 years), however, when the size of cellulose product was taken into consideration, the most excellent narrowest particle size of nanocellulose was obtained from HPH (MechCN-C2 process) on yielding CNF at the diameter ~ 3–7 nm^68^. Moreover, the price of nanocellulose is extremely higher than microcrystalline cellulose as CNF has been versatilely used as functionalized 2D nanomaterial in numerous applications. Hence, MechCN-C1 and MechCN-C2 processes are of interest for scaling up to produce CNF in a larger scale.

The fixed capital and utility cost for ultrasonication (MechCN-C1 process) and HPH (MechCN-C2 process) processes were the majority among all costs (Table S6). To optimize the utility cost of HPH (MechCN-C2 process), a reduction of HPH cycle (less than 12 passes) is another way to reduce the overall costs of CNF production. Therefore, in the present work, the application of ultrasonic defibrillation and the optimization of HPH cycle were further investigated. It was evaluated that the reduction of HPH cycles from 12 to 4 passes or 8 passes will substantially reduce utility cost and thus enhance NPV and IRR to similar level as other processes, and the payback period will be possibly reduced to 3–4 years accordingly.

### Experiments on nanocellulose production from EFB based on the selected most feasible process

Based on the process design in the previous section, cellulose was extracted from raw EFB by using various ChCl based DESs at different temperatures (60, 80 and 100 °C) at atmospheric pressure for 8 h. After bleaching, the size of cellulose was mechanically reduced by ultrasonication and HPH at different cycles (passes) from 4 to 12 passes at a constant pressure of 150 MPa.

#### Cellulose extraction by DESs

**Figure 1:**
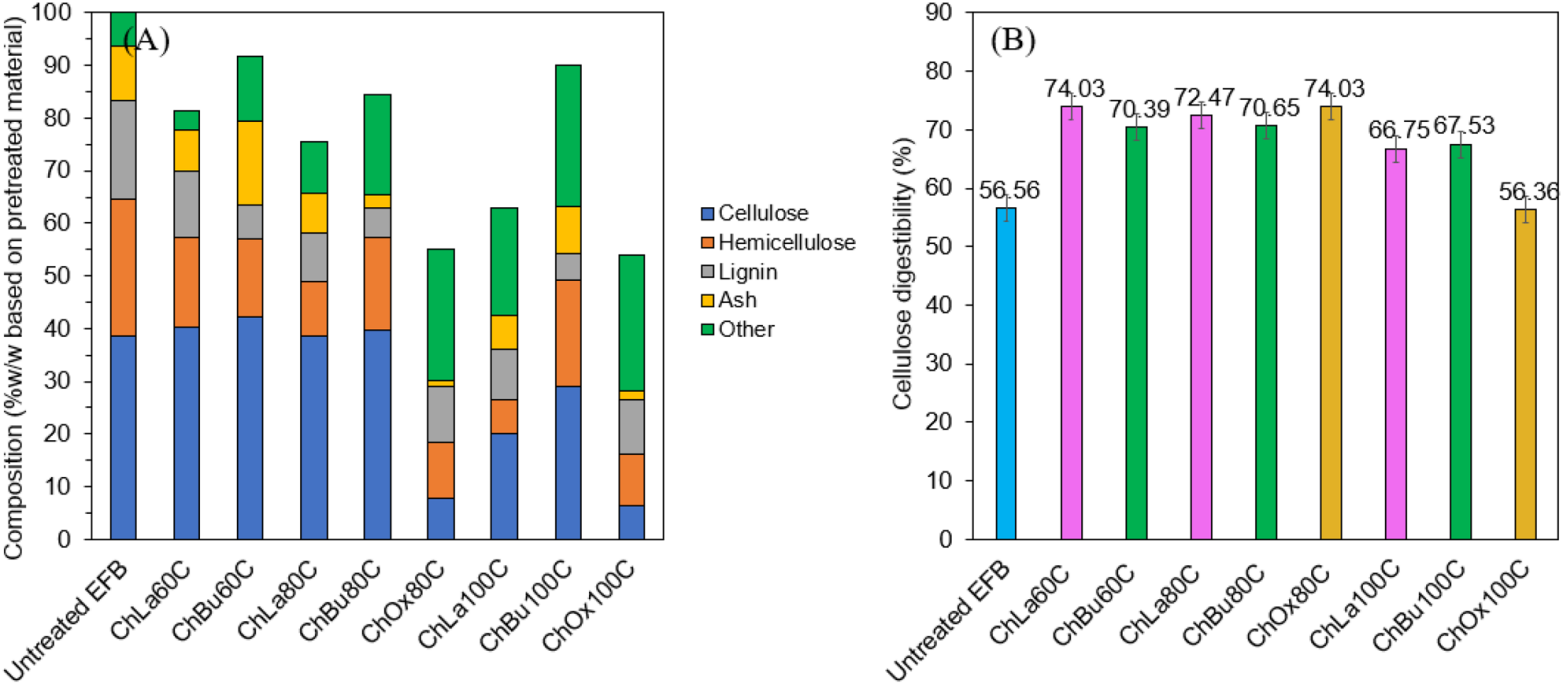
(**A**) Compositional analysis of pretreated EFBs in different DES and temperature varied at 60 °C, 80 °C and 100 °C for 8 h under reflux, and (**B**) Cellulose digestibility of extracted cellulose in pretreated EFB.

The chemical composition of pretreated EFBs was analyzed and compared with raw EFB as shown in Fig. 1A. Cellulose content in raw EFB was 38.5% w/w and after pretreatment with DES, the maximum cellulose content in pretreated material was 42.3% w/w for ChBu pretreatment at 60 °C (ChBu60C) and 40.2%w/w from ChLa pretreatment at 60 °C (ChLa60C), respectively. In case of ChLa pretreatment, an increased temperature from 60 to 80 °C and 100 °C resulted in a substantial removal of hemicellulose in a rising degree dependent on elevated temperature. Hemicellulose removal of 33.9%w/w, 60.8%w/w and 75.5%w/w based on hemicellulose in raw EFB were observed from ChLa60C, ChLa80C and ChLa100C, respectively while lignin removal was detected at 33.1%w/w, 49.6%w/w and 48.5%w/w based on lignin in raw EFB, respectively. Lignin removal was highest at 80 °C (ChLa80C), and an increased temperature to 100 °C (ChLa100C) did not show any improvement. ChLa80C contained highest cellulose content at 43.56%w/w along with 10.24%w/w hemicellulose, 9.48%w/w lignin and 7.46%w/w ash (Fig. 1A).

Similar results were found in case of ChOx pretreatment where hemicellulose removal was 59.6%w/w and 63.1%w/w from ChOx80C and ChOx100C, respectively while lignin removal was reported as 43.4%w/w and 44.9%w/w, respectively. Hemicellulose was degraded in higher extent relative to lignin due to acid constituents namely lactic acid (ChLa) and oxalic acid (ChOx) as hydrogen bond donors in DES solvent. In a study, pretreatment and enzymatic hydrolysis of rape straw were carried out by Li et al. using DES based on choline chloride and oxalic acid. The effects of DES treatment on the chemical composition, structural alteration, and enzymatic saccharification of RS were investigated. They reported that DES with a low molar ratio of HBA to HBD (ChCl:Ox 1:3) showed the greatest viscosity as 80.7 mPa⋅s, leading to low pretreatment efficiency. An increase of ChCl:Ox ratio from 1:3 to 3:1 mol/mol significantly reduced viscosity of DES solution from 80 to 18.1 mPa⋅s, therefore the mass transfer could be better at higher ChCl:Ox ratio. Interestingly, at moderate temperature between 110–120 °C, ChCl:Ox ratio of 1:2 mol/mol showed the best delignification and enzymatic saccharification efficiency as 76% and 89%, respectively. Further increase of Ox to ChCl in the DES (ChCl:Ox of 1:3) gave adverse effect on the efficiency of enzymatic saccharification. This phenomenon could be attributed to the excessive removal of xylan and lignin, leading to a higher likelihood of glucan aggregation. Consequently, this resulted in decreased accessibility of cellulose and reduced efficiency of enzymatic digestion. Nevertheless, at more severe temperature between 130–140 °C, ChCl:Ox ratio of 2:1 and 3:1 mol/mol performed better and exhibited on 81.5–87.5% delignification and 95.1–95.5% saccharification efficiency. The study demonstrated that the ratio of choline chloride and oxalic acid ratio is important in the removal of lignin and extraction of cellulose from lignocellulose materials^70^. In addition, the characteristics of DESs can be measured using the Kamlet-Taft parameters. The parameters α, β, and π* in this approach describe the hydrogen bond acidity, basicity and polarity of a solvent^71^. The α and β play a crucial role in enhancing the effectiveness of pretreatment^72^. DES with a high α value exhibits easy release of H^+^, whereas DES with a high β value has a greater capacity to receive H^+^. The large α, β, and π* values of DES serve as an important part in the decomposition rate of xylan and lignin in biomass pretreatment^73,74^. The basicity value in this study is equivalent to that of choline chloride, while the acidic characteristics exhibit variation. Oxalic acid has the greatest H^+^ release, followed by lactic acid, and the lowest is found in 1,3 butanediols.

Figure S7 exhibited the cleavage product of cellulose to water-soluble cello-oligomer in ChOx and ChLa, however cello-oligomer was not found in ChBu. ChOx showed greater extent of cellulose hydrolysis to cellobiose at 11.3%w/w and 24.4%w/w from ChOx80C and ChOx100C, respectively. The result was in good agreement of low cellulose content in ChOx pretreated material as only 9.88%w/w and 6.47%w/w based on treated EFB from ChOx80C and ChOx100C (Fig. 1A), respectively. This was owing to considerably too severe hydrolysis condition of cellulose catalyzed by oxalic acid that caused depolymerization of cellulose to short chain oligosaccharides which were soluble in aqueous solution. The results were in good accordance with a previous report revealing that significant cleavage of cellulose chains to nanocrystal was observed in ChOx solvent^57^ and low fiber yield was obtained.

In case of ChBu pretreatment, lignin was more selectively removed compared with hemicellulose. Lignin removal of 65.2%w/w, 69.6%w/w and 73.5%w/w based on lignin in raw EFB was detected from ChBu60C, ChBu80C and ChBu100C, respectively corresponding to lignin content in residual cellulose of 6.55%w/w, 5.71%w/w and 4.99%w/w in pretreated EFB (Fig. 1A) while hemicellulose removal was calculated as 43.9%w/w, 32.8%w/w and 22.8%w/w based on hemicellulose in raw EFB, respectively. An increase of temperature, substantially enhanced lignin removal, on the other hand decreased hemicellulose removal. This was possibly due to the high polarity of ChBu solvent that was reported to be as same as ethanol solvent when the Nile Red Polar Parameter values (E_NR_) of ethanol and DES were calculated from UV–Vis absorbance after adding Nile Red Dye into each DES investigated^75^. This observation indicates that the spatial arrangement of 1,3-propanediol’s molecules is favorable for creating a hydrogen-bonded complex with ChCl^76^ compared with other polyalcohols such as 1,4-butanediol^77^, 1,2-propanediol, 1,3-propanediol, and 1,2-butanediol as hydrogen bond donors^76^. Consequently, ChBu could extract lignin from EFB structure with higher efficiency relative to other DESs investigated *i.e.,* ChLa and ChOx. As lignin and hemicellulose are formed in a lignin-carbohydrate complexes, therefore hemicellulose removal was also high for ChBu extraction, and thus led to low content of lignin and hemicellulose in ChBu pretreated samples as shown in Fig. 1A.

However, when cellulose content in pretreated material was taken into consideration, ChLa and ChBu pretreatment at 60 °C and 80 °C provided high cellulose content while lignin and hemicellulose were selectively removed. ChLa and ChBu pretreatment at all temperatures yielded relatively high cellulose content that meant cellulose structure was intact. At 80 °C of pretreatment, cellulose contents in ChLa80C and ChBu80C were 43.56%w/w and 37.65%w/w, respectively. It was obvious that ChBu80C was still composed of hemicellulose and lignin greater than those of ChLa80C. The cellulose content of ChLa100C and ChBu100C were considerably reduced to 28.09%w/w and 30.09%w/w based on pretreated EFB, respectively. Therefore, cellulose destruction was obtained at elevated temperature from 60 to 100 °C and thus cellulose content decreased from 40–42%w/w to 29–30%w/w for ChLa and ChBu as demonstrated in Fig. 1A. Therefore, ChLa and ChBu were suitable pretreatment methods for nanocellulose production, and the final yield of cellulose as well as characteristics of nanocellulose were used to select the most suitable process for nanocellulose production.

Enzymatic digestibility of pretreated EFBs was analyzed for hydrolyzable sugar released into supernatant and reported as cellulose digestion ability as shown in Fig. 1B. From Fig. 1B, the performance of DESs pretreatment can best describe the value of cellulose digestibility^78^. ChLa obtained a fulfilling cellulose digestibility from the pretreatment at 60, 80 and 100 °C which were 74.03%, 72.47%, and 66.75%, respectively. Cellulose digestibility of the pretreated EFB by ChBu solvent at the reaction temperature of 60, 80 and 100 °C were 70.39%, 70.65% and 67.53%, respectively. ChOx80C pretreated EFB achieved the maximum cellulose digestibility as 74.03%, although low cellulose content was found in pretreated EFB due to cleavage of cellulose to soluble cello-oligomers. On the contrary, ChOx100C gave lowest cellulose digestibility as 56.36% since relatively high percentage of lignin was remained in the material. An enhanced pretreatment temperature of acidic DESs (ChLa and ChOx) on EFB pretreatment considerably caused high amount of lignin content in pretreated material which hindered the susceptibility of cellulase and hemicellulase enzymes to hydrolyze cellulose and hemicellulose^53^. Lignin adsorption onto binding sites of enzyme was the main reason of lower cellulose hydrolysis efficiency regarding non-productive binding. Besides, the presence of lignin in biomass obstructs the efficient adsorption of cellulase by acting as a shield around the cellulose, thus impeding the access of cellulases^79^. Hence, one of the reasons that influences cellulose digestibility is the ability to dissolve cellulose^80^ and acidic DESs was proven to have a higher biomass structural disruption compared to other DESs which enhanced the cellulose accessibility that influenced cellulose digestibility^78^. Consequently, the result demonstrated that the pretreatment of EFB with ChLa80C for 8 h was a suitable condition for EFB pretreatment and cellulose digestibility.

Considering pretreated EFBs obtained from the same DESs, cellulose yield apparently diminished with an increasing of reaction temperature as demonstrated in Fig. 1A. The reaction temperature of 80 °C was confirmed as a suitable pretreatment temperature compared with 100 °C. Pretreated EFBs from ChLa and ChBu at the reaction temperature of 60 °C were also obtained a satisfied amount of cellulose content. Whereas, the impurities of hemicellulose and lignin were highly remained in pretreated EFBs. The optimization of pretreatment process revealed that harsh conditions, such as prolonged reaction time and excessively high operating temperatures, could potentially cause thermal degradation of the DES compounds. These factors should be taken into account to maintain the stability and effectiveness of the DES during the delignification process^43^. A similar temperature dependence was observed in the study investigating the extraction mechanism of cellulose using ChCl-based DESs. The cellulose was extracted in the DES under different reaction temperatures, and it was found that the extraction process exhibited a temperature-dependent behavior. This suggests that the temperature plays a crucial role in the extraction of cellulose in ChCl-based DESs. The result demonstrated that a high temperature corresponded to a strong ability of the solvent to delignification and some destructive cellulose removal^81^.

#### Bleaching of extracted cellulose from EFB

**Figure 2:**
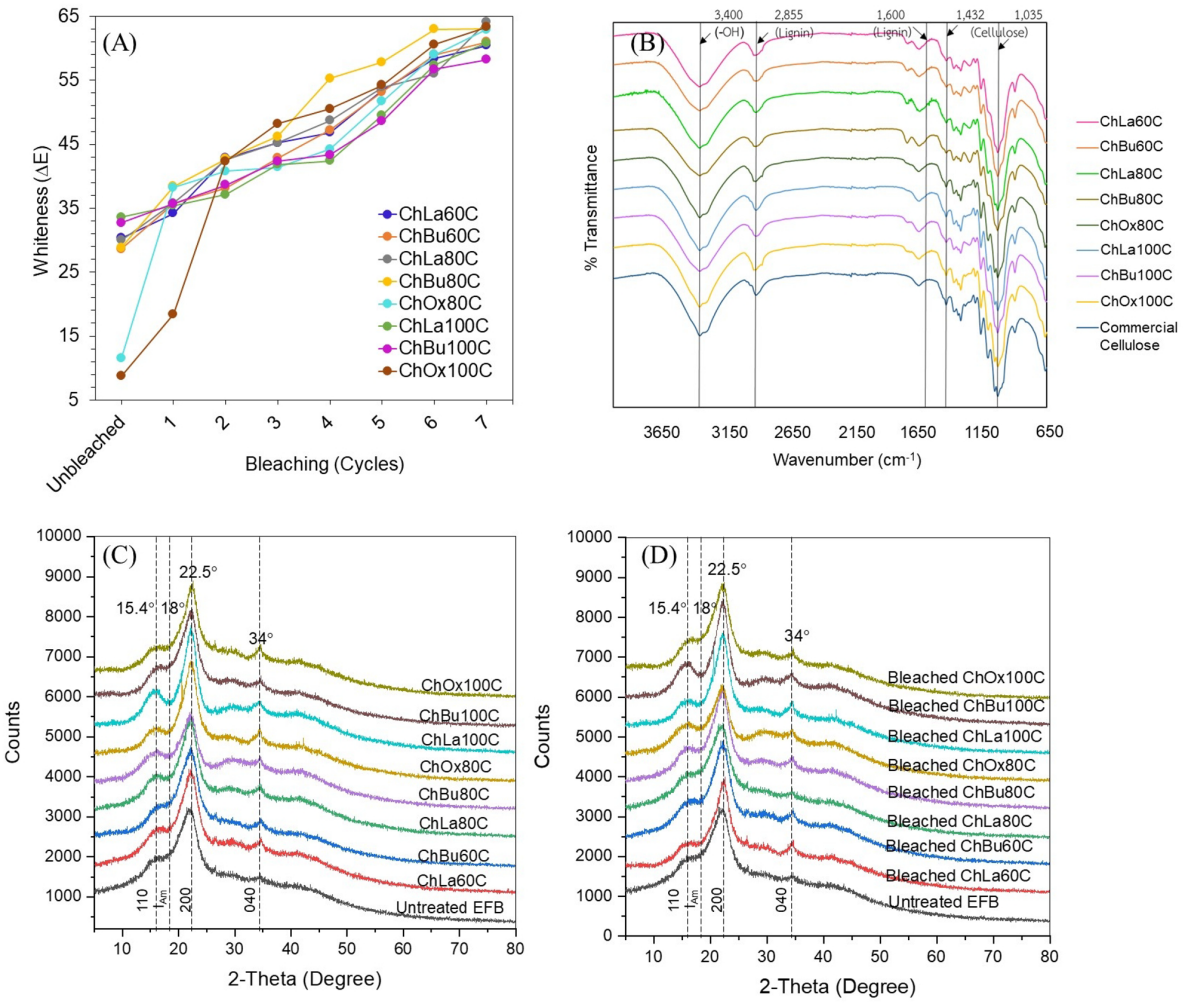
(**A**) Change of whiteness (ΔE) of bleached cellulose at different cycles of bleaching, (**B**) FTIR spectra of bleached cellulose, (**C**) XRD patterns of pretreated EFB, and (**D**) XRD patterns of 7th cycle of bleached cellulose extracted from EFB using different DES pretreatments at 60 °C, 80 °C and 100 °C for 8 h.

Pretreated EFBs from all DESs were bleached with 1.5 wt% NaClO_2_ and acetic acid solution repeatedly for seven cycles until the whiteness reaching the same level as commercial cellulose at which ΔE was 57 as demonstrated in Fig. S8. The results showed that the whiteness of all extracted cellulose after 6–7 times of bleaching was as high as commercial cellulose. Figure 2A additionally showed the changing rate of cellulose whiteness for each cycle of bleaching. ChOx pretreatment at 80 °C (ΔE = 12) and 100 °C (ΔE = 9) gave darkest color of pretreated material before bleaching, nevertheless a substantial enhancement of whiteness to the same level as other DESs extracted cellulose was observed at only the 1st and 2nd cycle of bleaching for ChOx80C and ChOx100C, respectively. At 80 °C of pretreatment, ChLa80C and ChBu80C provided highest whiteness value beyond commercial cellulose (ΔE = 57) as ΔE = 64.1 and ΔE = 63.1, respectively. The white color of cellulose could indicate a lower amount of lignin content in cellulose that makes them appeared white after bleaching^49^. The process of NaClO_2_ delignification generates chlorine dioxide (ClO_2_) under acidic conditions. This ClO_2_ then acts to break the ester bond between lignin and hemicellulose, as well as the ether and carbon–carbon bonds in lignin molecules^82^. From Table 1, the result concluded that seven-cycle bleaching was suitable for DESs extraction of cellulose from EFB at 60–100 °C for 8 h. After bleaching, ChLa80C provided the highest cellulose yield of 26.37%w/w based on EFB or 67.12%w/w cellulose yield based on cellulose in raw EFB (Table 1). Since the overall process steps after DES pretreatment (bleaching, ultrasonication and HPH) are the same for all DESs, thus OPEX is solely influenced by chemical cost of DESs based on the yield of bleached cellulose production in different DES. As shown in Table S7, the best bleached cellulose yield from Table 1 were selected for each DESs for the calculation of economic analysis (6.80% cellulose for ChOx80C, 50.30% cellulose from ChBu60C, and 68.50% cellulose for ChLa80C). When the DESs cost is computed based on 10 g raw EFB and compared with the amount of bleached cellulose produced, although ChBu60C provided lower amount of cellulose of 1.94 g compared with ChLa80C that could generate 2.64 g cellulose from 10 g EFB, lowest cost of cellulose as 1.75 USD/g cellulose was achieved from ChBu60C compared with 2.09 USD/g cellulose from ChLa80C. This was because the molar ratio of ChCl:lactic acid and the cost of lactic acid which is higher than that of 1,3-butanediol as shown in Table S7. Moreover, ChOx80C gave lowest cellulose yield and greatest DES cost. Although, DES cost of ChLa was greater than ChBu, however a significant reduction of OPEX could be obtained from DES recycling and the optimization of HBA-to-HBD ratio at specific pretreatment temperatures^70,83^. Therefore, both ChLa80C and ChBu60C were seemingly suitable for the further mechanical nano-defibrillation steps, ultrasonication and HPH, for nanocellulose production.

**Table 1:** Crystallinity index (%CrI) of cellulose extracted from different DES pretreatments and after the 7th cycle of bleaching, and cellulose yield after the 7th cycle bleaching.

Sample	CrI (%)		Cellulose content in 7th cycle bleached samples (%w/w)	Cellulose yield after bleaching (%w/w based on EFB)	Cellulose yield after bleaching (%w/w based on cellulose in raw EFB)
	After DES pretreatment	After 7th cycle of bleaching			
Untreated EFB	30.02		NA	38.50	100.0
ChLa60C	36.35	47.09	72.56	23.54	61.1
ChBu60C	35.4	49.74	74.14	19.37	50.3
ChLa80C	37.94	52.24	88.96	26.37	68.5
ChBu80C	31.21	50.15	86.41	13.16	34.2
ChOx80C	36.68	55.32	84.27	2.61	6.8
ChLa100C	42.56	57.34	73.54	11.08	28.8
ChBu100C	36.85	55.26	71.64	13.17	34.2
ChOx100C	31.24	51.92	85.36	1.66	4.3

Figure 2B shows FTIR spectroscopy of bleached cellulose compared to commercial cellulose. The results revealed the high absorption at wavenumber 3400 cm^−1^ representing the − OH functional group, the peak at wavenumber 1035 cm^−1^ attributed to the CO stretching, and the peak at wavenumber 1432 cm^−1^ associated with the − CH_2_ vibration of cellulose. The mentioned FTIR peaks are corresponding to cellulose peaks appeared on eco-friendly extraction of cellulose from EFB using formic acid and hydrogen peroxide under mild condition^49^. Nazir and colleagues found similar peaks suggesting the C–H stretching of OCH_3_ at the wavenumber 2855 cm^−1^ and the peak at the wavenumber 1600 cm^−1^ representing lignin’s C=C functional group. The FTIR spectra demonstrate that the lignin peak at the wavenumber 1600 cm^−1^ was absent in all bleached samples. The removal of lignin can be attributed to the reaction between NaClO_2_ and lignin, which leads to aromatic substitution and scission, along with the displacement of lignin side chains, particularly in an acidic environment^84^. Additionally, the Cl^−^ ions that remain can form hydrogen bonds with the hydroxyl groups (OH) in the polysaccharide, which aids in the separation of lignin-polysaccharide complex^85^. It was reported that the inclusion of acidic DES residue (i.e., ChLa and ChOx) in pretreated wet sample prior to bleaching may additionally assist in the promotion of NaClO_2_ delignification^86^.

The crystallinity of cellulose (CrI) is an important factor influencing the hydrolysis capacity of lignocellulose. Lignin and hemicellulose both contain amorphous regions. The XRD data of untreated and DES pretreated lignocellulose materials are shown in Fig. 2C. X-ray diffraction analysis of untreated EFB exhibited two prominent peaks in the 2θ angle range, approximately at 15.4° and 22.5°. The first peak arose from an overlapping signal contributed by the 110 plane of cellulose, while the second peak corresponded to the 200 plane of cellulose^87^. The CrI of raw EFB was 30.02% and an obviously enhanced CrI was detected after DES pretreatment of EFB in all samples since amorphous moieties (I_Am_ at 18°) composed mainly of lignin and hemicellulose were removed as shown in Table 1. Similar XRD patterns of 7th-cycle bleached cellulose was demonstrated in Fig. 2D. After bleaching, a markedly increase of %CrI from ca. 31–42% to ca. 47–57% was observed as demonstrated in Table 1. Similar XRD results were reported by Li et al., when rape straw (RS) was treated with DES, ChCl:Ox. The reported XRD peaks was presented at the 2*θ* of 15.0°, 22.5° and 31.5°. The crystallinity was increased significantly as 66.7% for DES-treated RS as compared to untreated RS which exhibited crystallinity of 35.4%. According to the report, the amorphous area of cellulose can be reduced by degradation through the action of DES, produced by ChCl:Ox^70^. In another study on ChCl/formic acid and ChCl/1,4-butanediol DES pretreatment of industrial xylose residue, CrI of ChCl/1,4-butanediol treated samples at 80, 100 and 120 °C for 2 h containing 71.8 to 77.3% cellulose and lignin from 13.5 to 20.3% was reported between 18.3 to 18.6% which was little greater than untreated sample (CrI = 13.3%). In contrast, ChCl/formic acid (1:1.5 mol/mol) increased CrI for 2-time between 27.8 and 29% in the same DES pretreatment condition when the sample contained 76.4–79.3% cellulose and 10.7–13.5% lignin^88^.

Previous research has demonstrated that the native cellulose found in biomass possesses a semi-crystalline structure known as cellulose-I_β_. In this structure, parallel chains of the glucan polymer are arranged together, forming flat sheets that are held together by inter-hydrogen linkages^89^. Typically, various cellulose lattice planes such as the plane 1–10 observed at diffraction angle 15.4°, the plane 110 at 16.2°, the plane 004 at 34°, the plane 021 at 22.1°, and the plane 200 at 22.5°^90^ are observed in the cellulose-I_β_ form, which is commonly found in higher plants^91^. However, commercial microcrystalline and nanocellulose primarily consist of the lattice plane 200 while cellulose lattice planes 1–10, 110, and 004 are comparatively less abundant than the aforementioned plane^90^.

#### High-pressure homogenization of ultrasonicated cellulose for nanocellulose production

**Figure 3:**
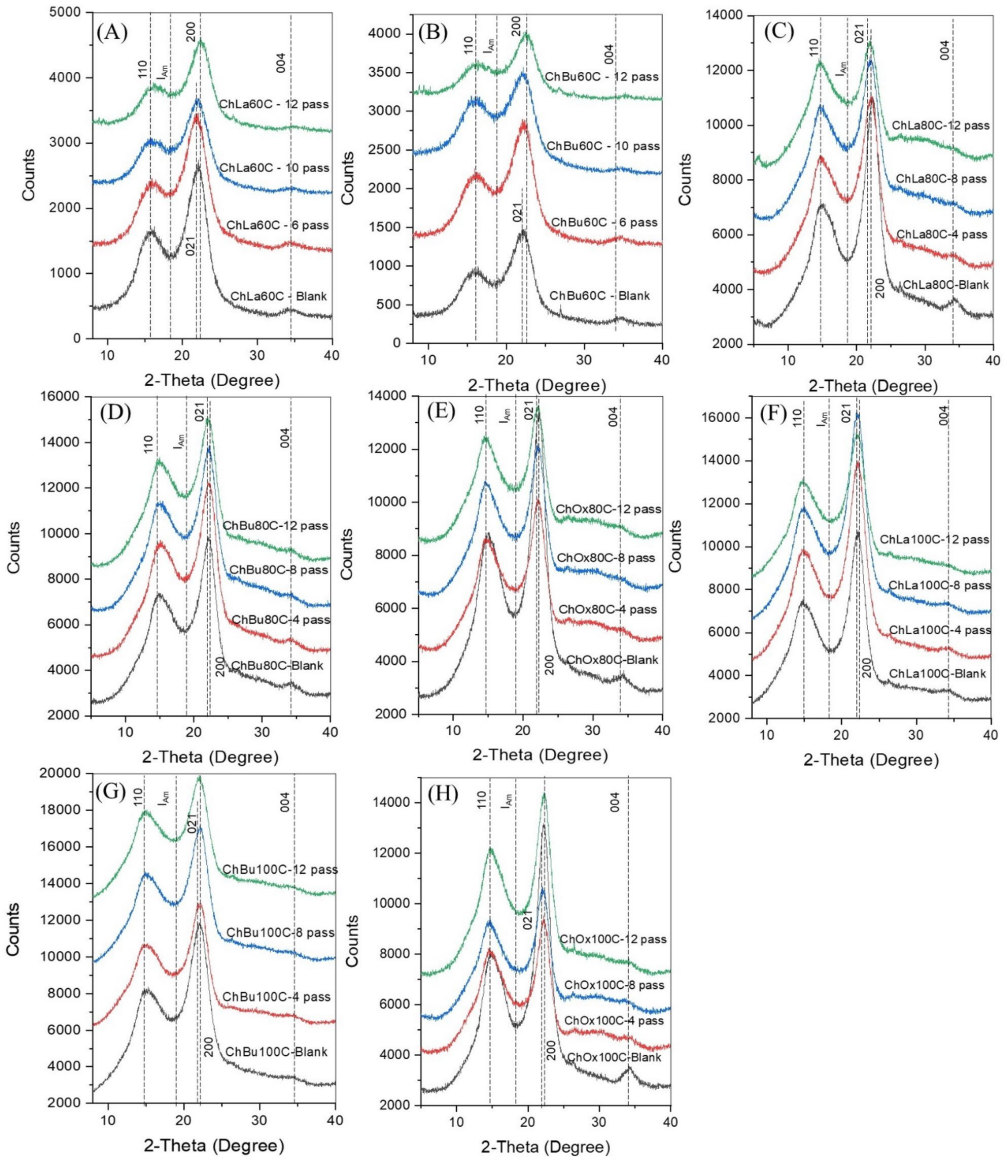
XRD diffractograms of nanocellulose after high-pressure homogenization (HPH) of bleached/ultrasonicated cellulose (Blank) for 4 passes, 8 passes and 12 passes at 150 MPa from extracted cellulose from (**A**) ChLa60C, (**B**) ChBu60C, (**C**) ChLa80C, (**D**) ChBu80C, (**E**) ChOx80C, (**F**) ChLa100C, (**G**) ChBu100C, and (**H**) ChOx100C using DES pretreatment methods for 8 h.

Ultrasonication of bleached cellulose caused fibrillation of fiber, and afterward high-pressure homogenization (HPH) process was applied to produce nanocellulose. The HPH forces occurred by rapidly alternating the opening and closing of the valve that contributes to a substantial level of fibrillation, promoting the formation of nanofibers^92^. From the XRD diffractograms of HPH nanocellulose at different passes in Fig. 3, cellulose lattice planes were obviously exhibited as followed: the plane 1–10 observed at diffraction angle 15.4°, the plane 110 at 16.2°, the plane 004 at 34°, the plane 021 at 22.1°, and the plane 200 at 22.5°^90^. These XRD peaks represented the structure of cellulose-I_β_ form, which is commonly found in higher plants^91^. Compared with untreated EFB from Fig. 2C,D, the DES delignification, bleaching and ultrasonication process provided nanocellulose with the strong XRD intensity of the 110 planes of the cellulose lattice. In the analysis of the HPH-nanocellulose sample's crystalline planes, the 200 plane was highly identified especially for the ChBu100C and ChOx100C as shown in Fig. 3G, and H. However, there was a slightly shift of the 021 plane and the 200 plane when applying HPH beyond 6 and 8 passes in case of ChLa60C, ChBu60C, ChLa80C, ChBu80C, ChOx80C, and ChLa100C as demonstrated in Fig. 3A–F. These discrepancies in detecting various crystalline lattice planes in nanocellulose samples may be attributed to the difference in the hydrogen-bond intensity (degree of organization) between the adjacent cellulose chains in those samples. Alteration between 021 and 200 planes was also observed in a previous study on DES pretreatment of rice straw^90^. Typically, compared to either 200 or 021 planes, the cellulose lattice planes 1–10, 110, and 004 have a relatively lower frequency of occurrence.

CrI of HPH nanocellulose from acidic DES pretreatment of EFB at 80 °C and 100 °C namely ChLa80C, ChOx80C, ChLa100C, and ChOx100C was found to be reduced slightly when increasing the number of passes as shown in Fig. S9. It was apparent that ultrasonication of acidic DES already enhanced CrI to the highest degree especially in the case of ChOx80C, ChLa100C and ChOx100C which provided 68.9, 73.5, and 74.7% CrI of 0-pass HPH nanocellulose samples, respectively. After 12-pass of HPH, CrI of the aforementioned nanocellulose (ChOx80C, ChLa100C and ChOx100C) was decreased to 64.0, 68.7 and 66.7% CrI (Fig. S9), respectively. An increased number of passes to 12th cycle substantially decreased crystallinity of nanocellulose observed by a reduction of the plane 200 in XRD pattern from Fig. 3. It can be inferred that beyond a certain number of homogenization cycles, excessive peeling would noticeably decrease the crystallinity of the cellulose nanofibers (CNF). Similar findings were reported when HPH parameters (pressure and number of cycles) were optimized on the crystallinity, isolated yield, and diameter of nananocellulose^93^. An increased number of passages through the homogenizer results in a higher energy imparted to the suspension, gradually breaking down the hydrogen bonds that hold the fibers together, and consequently increasing fibrillation efficiency^94^.

The optimal number of passes for HPH of ChBu pretreated EFB was somewhat different. At low temperature of ChBu pretreatment at 60 and 80 °C, ChBu60C and ChBu80C required 4-pass HPH and 8-pass HPH to enhance the CrI of nanocellulose up to 65.8 and 58.5%CrI, respectively. Similar results were revealed for a significant reduction of crystallinity of nanocellulose when the pressure and number of passes of HPH process increased^93^. The observed increase in crystallinity as the number of homogenization cycles increases from the blank (ultrasonicated bleached cellulose) to 4th–8th pass of HPH process may be attributed to the rearrangement of crystals within the water medium. The mechanical process has the ability to rearrange the restricted sections of the nanofibers, which were originally disordered due to the nanocrystal's or nanofibers' increased mobility in water^95^.

**Figure 4:**
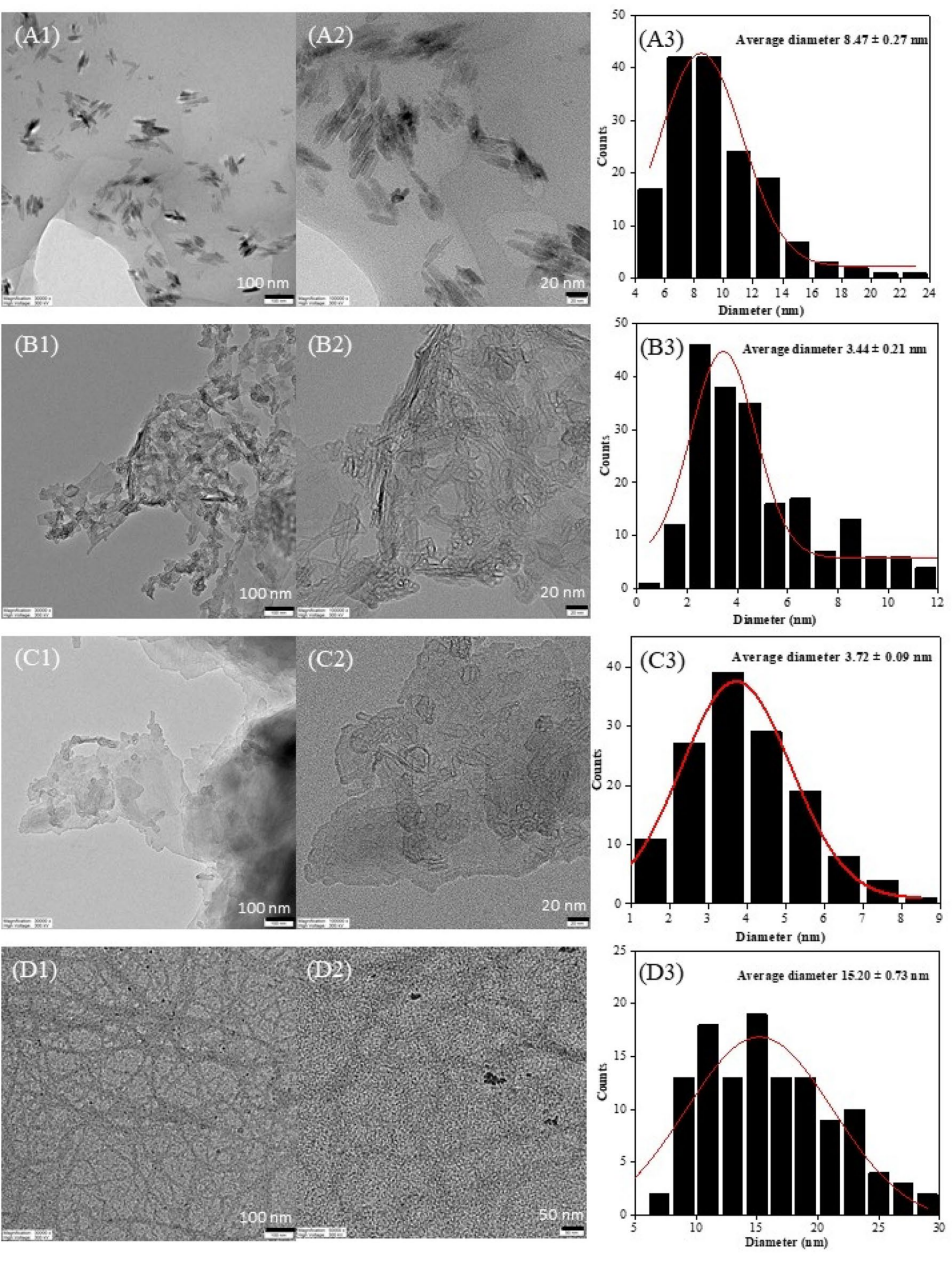

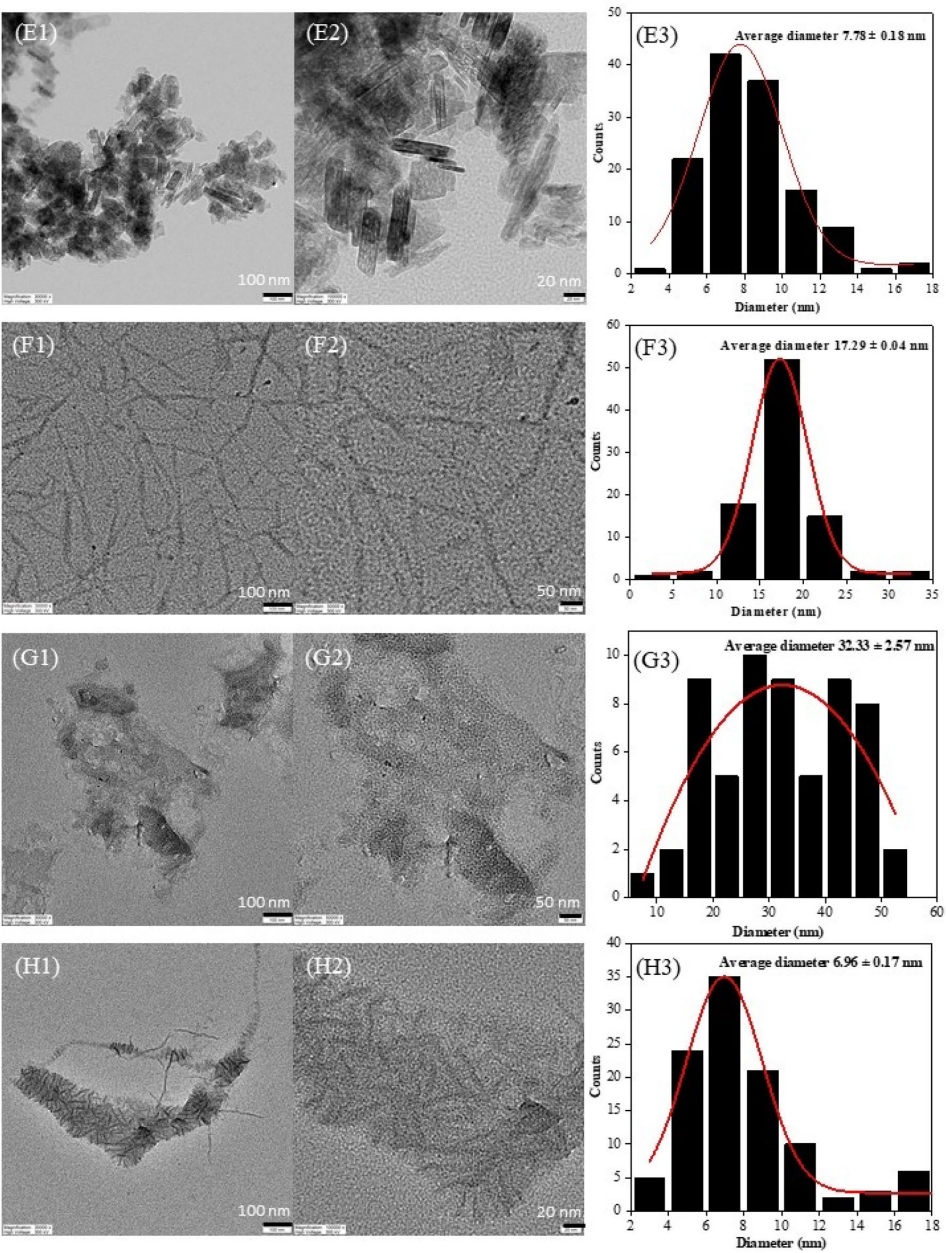
FETEM images: image (1) 30,000× magnification, and (2) 50,000× or 100,000× magnifications, and (3) histogram presenting nanocellulose size distribution from 12-pass HPH process at 150 MPa of different DES pretreated samples; (**A**) ChLa60C, (**B**) ChBu60C, (**C**) ChLa80C, (**D**) ChBu80C, (**E**) ChOx80C, (**F**) ChLa100C, (**G**) ChBu100C, and (**H**) ChOx100.

In case of ChBu100C, the greatest CrI was achieved as 58.5% CrI without passing HPH (0-pass HPH), therefore at elevated temperature of ChBu100C pretreatment (Fig. S9), further ultrasonication for mechanical treatment is sufficient energy to fibrillate cellulose structure toward nanocellulose and not necessary to apply HPH. In details, elucidated morphological analysis is required to conclude the hypothesis. At 12-pass HPH, the highest CrI in the present study was obtained from ChLa100C (68.7%CrI) which was in good correspondence to TEM images (Fig. 4F).

From TEM image analysis, the average diameters of nanocellulose from different DES pretreatments followed by 7-cycle bleaching, ultrasonication and 12-pass HPH process were demonstrated in Fig. 4. The results confirmed that nanocellulose produced from all conditions displayed the diameter in a range of 3.44 nm to 32.33 nm. Interestingly, in case of ChBu100C, 12-pass HPH process was too severe to produce cellulose nanofiber, instead of that, the breakdown of cellulose chains to a cluster of soluble oligosaccharides were observed as shown in Fig. 4G. From the results, acidic DESs pretreatment of EFB namely ChLa and ChOx with 12-pass HPH could successfully produce cellulose nanocrystal or nanoparticles as illustrated in Fig. 4A for ChLa60C, Fig. 4E for ChOx80C, and Fig. 4H for ChOx100C corresponding to the average diameter of 8.47 ± 0.27 nm, 7.78 ± 0.18 nm, and 6.96 ± 0.17 nm, respectively. Most of acidic DES pretreatment (ChLa60C, ChOx80C, and ChOx100C) gave cellulose nanocrystals at an average diameter less than 10 nm and the length between 50 and 100 nm (Fig. 4) from which the aspect ratio or length-to-diameter ratio (L/d) was between 5 and 10. Nevertheless, in the present work, cellulose nanofibers were successfully produced from two conditions namely ChBu80C (Fig. 4D) and ChLa100C (Fig. 4F) when processed with ultrasonication and followed by 12-pass HPH. This gave the nanofibers with the average diameter of 15.20 ± 0.73 nm and 17.29 ± 0.04 nm, and the length of nanofibers in a range of more than 1000 nm and ~ 300–600 nm, respectively. The width and length of cellulose nanofibers treated with ChBu80C, displayed average diameter of 15.20 nm and average length of 1000 nm or L/d = ~ 65.8. For ChLa100C, it generated cellulose nanofibers with an average diameter and length of 17.29 nm and ~ 300–600 nm, respectively or L/d = ~ 17.4 to ~ 34.7 (Fig. 4). ChBu60C and ChLa80C followed by 12-pass HPH gave cellulose fiber with a clumpy structure containing lots of fiber aggregation as shown in Fig. 4B,C, respectively. A greater DES pretreatment temperature at 100 °C for ChLa100C treatment may cause harsh interruption of long fibers to short length after 12-pass HPH compared with more suitable ChBu80C pretreatment that was appropriate for 12-pass HPH to yield nanocellulose fibers. However, both ChLa100C and ChBu80C yielded aggregate nanocellulose fibers suitable for nanofiber production for liquid and air filter fabrication. Additionally, it has been reported that addition of nanocrystal fillers into nanofiber matrix significantly enhanced filtration efficiency. Consequently, adjustable of nanocellulose morphology is undoubtedly important and a challenging aim for the nanocellulose utilization in the future.

The summary of L/d ratios, morphology and viscosity tested at the sweeping frequency of 0.1 Hz of all nanocellulose samples was demonstrated in Table S8. Depending on their morphology, nanocellulose with clumpy structure gave substantially high viscosity of 147.9 Pa⋅s for ChBu60C and 46.65 Pa⋅s for ChLa80C. Subsequently lower viscosity in a range of 32.45 to 99.42 Pa⋅s was obtained from nanocrystal structure after passing HPH for 12 cycles namely ChLa60C (99.42 Pa⋅s), ChOx100C (41.1 Pa⋅s) and ChOx80C (32.54 Pa⋅s). In case of nanofiber structure, after 12-pass HPH relatively lower viscosity was shown between 12.92 and 14.40 Pa⋅s for ChBu80C and ChLa100C, respectively.

**Figure 5:**
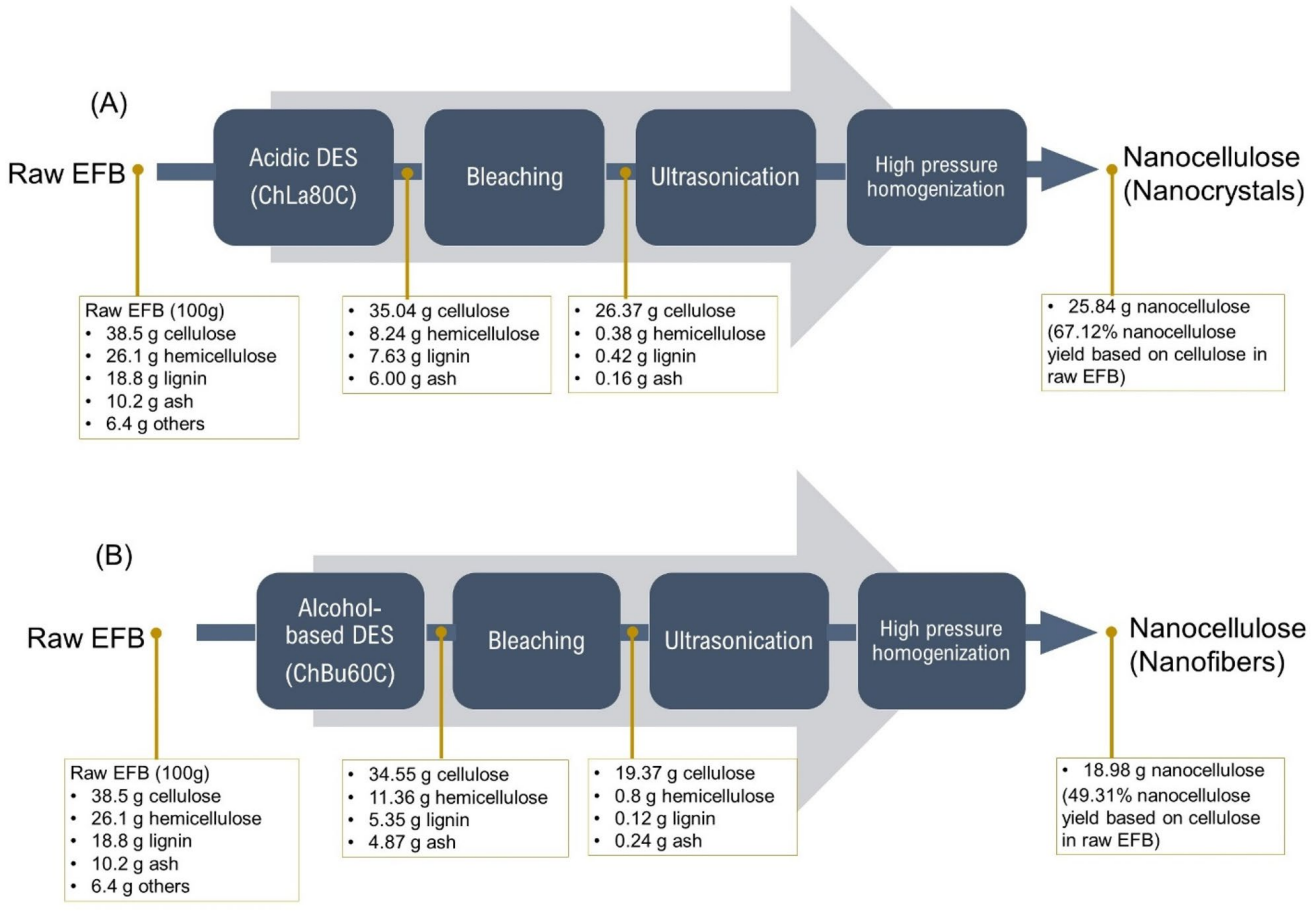
Mass balance of acidic-based DES (ChLa80C) and alcohol-based DES (ChBu60C) pretreatment for 8 h of raw EFB, followed by 7-cycle bleaching, ultrasonication at 20 kHz for 20 min, and 12-pass HPH at 150 MPa 30 °C for nanocellulose production.

Figure 5 illustrated the mass balance of acidic DES (ChLa80C) and alcohol-based DES (ChBu60C) pretreatment of raw EFB, followed by 7-cycle bleaching using sodium chlorite and acetic acid solution, ultrasonication at 20 kHz for 20 min, and 12-pass HPH at 150 MPa and 30 °C for nanocellulose production. Based on 100 g raw EFB which contains 38.5 g cellulose, the highest nanocellulose yield of 25.84 g was produced using ChLa80C process (Fig. 5A). This acidic DES substantially removed hemicellulose from 26.1 g in raw EFB to 8.24 g in pretreated EFB equivalent to 68.43% removal while lignin was removed as 59.41%. Massive loss of cellulose was found at bleaching process from 35.04 to 26.37 g corresponding to 24.74% cellulose loss. Final product which was cellulose nanocrystals with an average diameter less than 10 nm were produced at 25.84 g which was 67.12% nanocellulose yield based on mass of cellulose in raw EFB. From Fig. 5A, final nanocellulose product from ChLa80C contained highest cellulose purity of 96.48%, and small contaminants remained (1.39% hemicellulose, 1.54% lignin and 0.59% ash).

In case of nanocellulose from ChBu60C pretreatment (Fig. 5B), although lignin was greatly removed from raw EFB (18.8 g lignin) to 5.35 g lignin which was corresponded to 71.54% lignin removal, nevertheless dark color of pretreated EFB was obtained as illustrated in Fig. S8. Owing to the swollen structure of ChBu60C pretreated EFB, oxidation of color chromophore caused greater loss of cellulose after bleaching accounted as 43.94% cellulose loss during 7-cycle bleaching. Therefore, only 18.98 g nanocellulose was produced (nanofiber with diameter less than 15 nm) from 100 g raw EFB that was equivalent to 49.31% nanocellulose yield based on cellulose mass in raw material. From Fig. 5B, final nanocellulose product from ChBu60C contained cellulose purity of 94.35%, and small contaminants remained (3.90% hemicellulose, 0.58% lignin and 1.17% ash).

**Figure 6:**
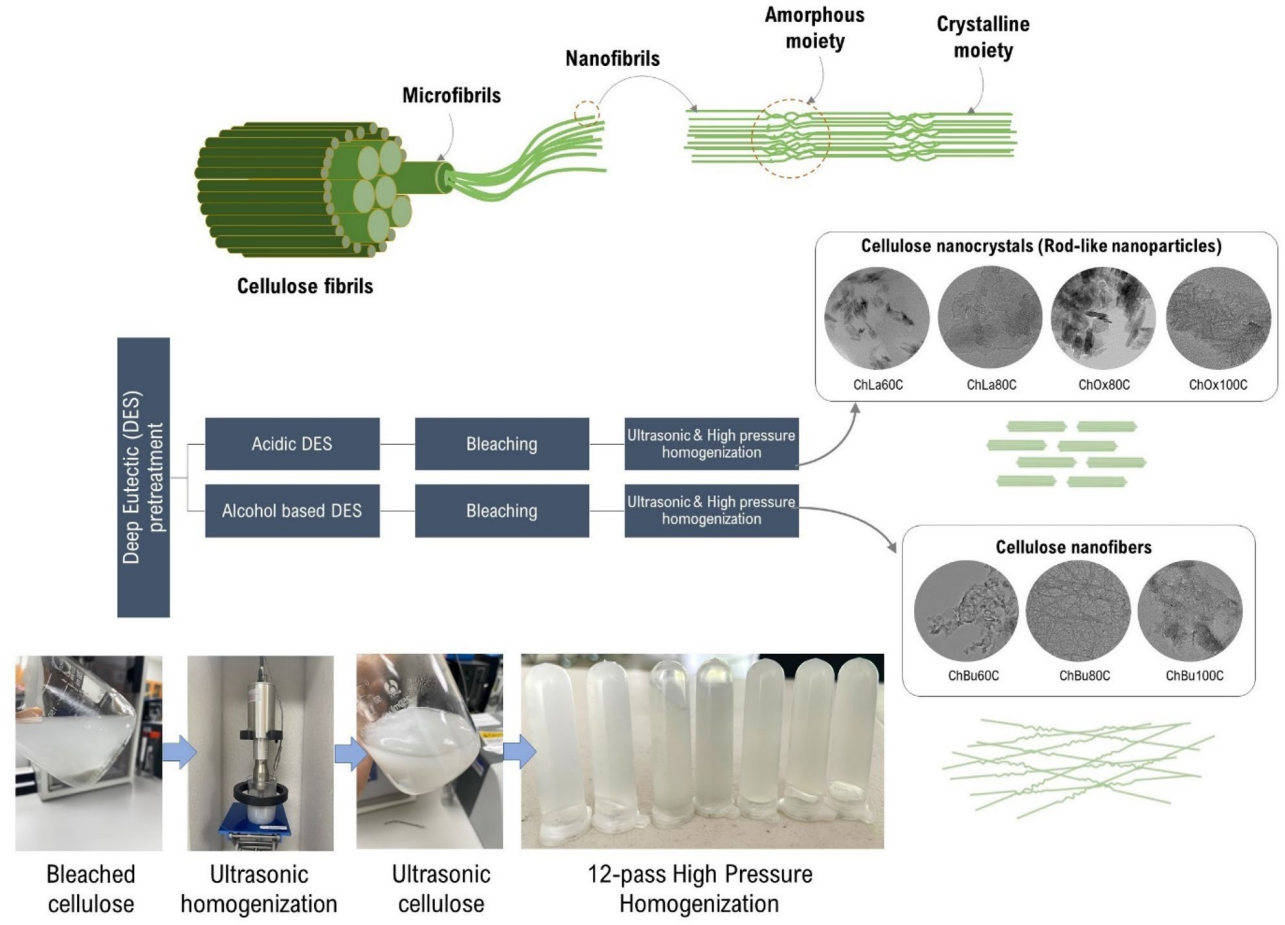
Illustration of acidic and alcohol based deep eutectic solvent (DES) pretreatment and subsequent nano-defibrillation using ultrasonic and high-pressure homogenization of EFB for nanocellulose production.

In conclusion, meaningfully highest nanocellulose yield (67.12% based on initial mass of cellulose in EFB) was achieved from ChLa80C pretreatment compared with other DES pretreatments investigated in the present study. Nevertheless, lower DES cost of ChBu60C for 16% relative to ChLa80C (Table S7) must be taken into account for further detailed feasibility analysis. Summary of acidic and alcohol based DES pretreatment of palm EFB followed by nano-defibrillation using ultrasonication and high-pressure homogenization of EFB for nanocellulose production was presented in Fig. 6. Most of nanorods and nano whiskers as well as clumpy morphology was obtained from acidic DESs while longer and interconnecting nanofibers was achieved from alcohol based DESs pretreatment. Further optimization of number of HPH cycle should be performed in order to obtain striking network of cellulose nanofibers or clumpy fibrous structure of nanocellulose for a specific purpose such as 3D printing, stabilizer, pickering emulsifier, etc.

## Conclusion

From the chemical engineering process design for nanocellulose production, the most proper process was selected for nanocellulose production from biomass in terms of minimum operating cost and highest nanocellulose yield. From the process design, DES extraction at low reaction temperature (<100 °C) followed by mechanical treatment using ultrasonication was the most promising. Therefore, the experimental section in the present work selected DES in a combination of ChCl as hydrogen bond acceptor and hydrogen bond donors were 1,3-butanediol (ChBu), oxalic acid (ChOx) and lactic acid (ChLa) for cellulose extraction. Ultrasonic fibrillation and subsequent high-pressure homogenization (HPH) with varying number of passes were a chosen equipment for the reduction of cellulose particle size. From the results, ChLa80C was the most optimal condition that could extract cellulose from palm empty fruit bunch at 35.04 g cellulose per 100 g of EFB. After bleaching, cellulose was defibrillated to nanofibers by using ultrasonication and subsequent 12-pass HPH. Massive production of nanocellulose as 25.84 g from 100 g EFB (67.12%w/w cellulose yield) was achieved within 18-h processing time from ChLa80C pretreatment. Although ChBu60C provided 49.31%w/w nanocellulose yield, however, 16% of DES cost of ChBu was lower than ChLa. Therefore, detailed feasibility analysis is necessary for further scaling up process. The findings additionally demonstrated different morphology of nanocrystals in nanorod-like structure from both ChLa60C and ChLa80C which was suitable for various application such as reinforcement filler in polymer matrix and formulation of molecular nanocarrier as well as in fabricating of magnetic, electronic, optical, and micromechanical devices. High purity of nanocellulose and tunable morphology as well as functionality produced makes this material applicable for high precision application.

### Supplementary Information

Supplementary Information.

## Data Availability

All data generated or analyzed during this study are included in this published article and its supplementary information files.

## References

[1] Omran AAB (2021). Micro- and nanocellulose in polymer composite materials: A review. Polymers.

[2] Gan PG, Sam ST, Abdullah MFB, Omar MF (2020). Thermal properties of nanocellulose-reinforced composites: A review. J. Appl. Polym. Sci..

[3] Ee LY, Yau Li SF (2021). Recent advances in 3D printing of nanocellulose: Structure, preparation, and application prospects. Nanoscale Adv..

[4] Ghilan A, Nicu R, Ciolacu DE, Ciolacu F (2023). Insight into the latest medical applications of nanocellulose. Materials.

[5] Lin N, Dufresne A (2014). Nanocellulose in biomedicine: Current status and future prospect. Eur. Polym. J..

[6] Ahankari SS, Subhedar AR, Bhadauria SS, Dufresne A (2021). Nanocellulose in food packaging: A review. Carbohydr. Polym..

[7] Giannelli R, Babudri F, Operamolla A, Thomas S, Pottathara YB (2021). Nanocellulose Based Composites for Electronics.

[8] Ji C, Wang Y (2023). Nanocellulose-stabilized Pickering emulsions: Fabrication, stabilization, and food applications. Adv. Colloid Interface Sci..

[9] Jaiswal AK (2023). Biodegradable cellulose nanocomposite substrate for recyclable flexible printed electronics. Adv. Electron. Mater..

[10] Gao L (2019). Flexible, transparent nanocellulose paper-based perovskite solar cells. npj Flexible Electron..

[11] Horta-Velázquez A, Morales-Narváez E (2022). Nanocellulose in wearable sensors. Green Anal. Chem..

[12] Trache D, Hussin MH, Haafiz MKM, Thakur VK (2017). Recent progress in cellulose nanocrystals: Sources and production. Nanoscale.

[13] Ai Y (2022). Toward cleaner production of nanocellulose: A review and evaluation. Green Chem..

[14] Rånby BG (1951). Fibrous macromolecular systems. Cellulose and muscle. The colloidal properties of cellulose micelles. Discuss. Faraday Soc..

[15] Dong XM, Revol JF, Gray DG (1998). Effect of microcrystallite preparation conditions on the formation of colloid crystals of cellulose. Cellulose.

[16] Araki J, Wada M, Kuga S, Okano T (1998). Flow properties of microcrystalline cellulose suspension prepared by acid treatment of native cellulose. Colloids Surf. Physicochem. Eng. Aspects.

[17] Camarero Espinosa S, Kuhnt T, Foster EJ, Weder C (2013). Isolation of thermally stable cellulose nanocrystals by phosphoric acid hydrolysis. Biomacromolecules.

[18] Turbak, A. F., Snyder, F. W. & Sandberg, K. R. Microfibrilated cellulose, a new cellulose product: properties, uses, and commercial potential. In *J Appl Polym Sci, Appl Polym Symp.* **37**, 815–827 (1983).

[19] Chen W (2011). Individualization of cellulose nanofibers from wood using high-intensity ultrasonication combined with chemical pretreatments. Carbohydr. Polym..

[20] Iwamoto S, Nakagaito AN, Yano H (2007). Nano-fibrillation of pulp fibers for the processing of transparent nanocomposites. Appl. Phys. A Mater. Sci. Process..

[21] Zimmermann T, Pöhler E, Geiger T (2004). Cellulose fibrils for polymer reinforcement. Adv. Eng. Mater..

[22] Zhang L, Tsuzuki T, Wang X (2015). Preparation of cellulose nanofiber from softwood pulp by ball milling. Cellulose.

[23] Jiang F, Hsieh Y-L (2013). Chemically and mechanically isolated nanocellulose and their self-assembled structures. Carbohydr. Polym..

[24] Jiang J, Zhu Y, Jiang F (2021). Sustainable isolation of nanocellulose from cellulose and lignocellulosic feedstocks: Recent progress and perspectives. Carbohydr. Polym..

[25] Jiang Y (2018). Effects of residual lignin on mechanical defibrillation process of cellulosic fiber for producing lignocellulose nanofibrils. Cellulose.

[26] Saito T, Nishiyama Y, Putaux J-L, Vignon M, Isogai A (2006). Homogeneous suspensions of individualized microfibrils from TEMPO-catalyzed oxidation of native cellulose. Biomacromolecules.

[27] Aulin C, Johansson E, Wågberg L, Lindström T (2010). Self-organized films from cellulose I nanofibrils using the layer-by-layer technique. Biomacromolecules.

[28] Liu P (2015). Nanocelluloses and their phosphorylated derivatives for selective adsorption of Ag+, Cu2+ and Fe3+ from industrial effluents. J. Hazard. Mater..

[29] Henriksson M, Henriksson G, Berglund LA, Lindström T (2007). An environmentally friendly method for enzyme-assisted preparation of microfibrillated cellulose (MFC) nanofibers. Eur. Polym. J..

[30] Liimatainen H, Visanko M, Sirviö JA, Hormi OEO, Niinimaki J (2012). Enhancement of the nanofibrillation of wood cellulose through sequential periodate-chlorite oxidation. Biomacromolecules.

[31] Xue B, Yang Y, Zhu M, Sun Y, Li X (2018). Lewis acid-catalyzed biphasic 2-methyltetrahydrofuran/H_2_O pretreatment of lignocelluloses to enhance cellulose enzymatic hydrolysis and lignin valorization. Bioresour. Technol..

[32] Reyes G (2019). Dissolution and hydrolysis of bleached kraft pulp using ionic liquids. Polymers.

[33] Smith EL, Abbott AP, Ryder KS (2014). Deep eutectic solvents (DESs) and their applications. Chem. Rev..

[34] New EK (2022). The application of green solvent in a biorefinery using lignocellulosic biomass as a feedstock. J. Environ. Manag..

[35] Wei X, Lin T, Wang L, Lin J, Yin X (2023). Research on deep eutectic solvents for the construction of humidity-responsive cellulose nanocrystal composite films. Int. J. Biol. Macromol..

[36] Loow Y-L (2018). Deep eutectic solvent and inorganic salt pretreatment of lignocellulosic biomass for improving xylose recovery. Bioresour. Technol..

[37] Tan YT, Chua ASM, Ngoh GC (2020). Deep eutectic solvent for lignocellulosic biomass fractionation and the subsequent conversion to bio-based products—A review. Bioresour. Technol..

[38] Zhu Y (2023). Acidic and alkaline deep eutectic solvents (DESs) pretreatment of grapevine: Component analysis, characterization, lignin structural analysis, and antioxidant properties. Int. J. Biol. Macromol..

[39] Loow Y-L (2017). Potential use of deep eutectic solvents to facilitate lignocellulosic biomass utilization and conversion. Cellulose.

[40] Majová V, Jablonský M, Lelovský M (2021). Delignification of unbleached pulp by ternary deep eutectic solvents. Green Process. Synth..

[41] Yong KJ, Wu TY (2023). Recent advances in the application of alcohols in extracting lignin with preserved β-O-4 content from lignocellulosic biomass. Bioresour. Technol..

[42] Wang X (2022). Dissolution and degradation of cellulosic fiber in carboxylic acid choline chloride-based deep eutectic solvents. Wood Sci. Technol..

[43] Liu Q (2019). Choline chloride-lactic acid deep eutectic solvent for delignification and nanocellulose production of moso bamboo. Cellulose.

[44] Li P (2022). Comparison of the degradation performance of seven different choline chloride-based DES systems on alkaline lignin. Polymers.

[45] Chen W (2015). Revealing the structures of cellulose nanofiber bundles obtained by mechanical nanofibrillation via TEM observation. Carbohydr. Polym..

[46] Bi W, Tian M, Row KH (2013). Evaluation of alcohol-based deep eutectic solvent in extraction and determination of flavonoids with response surface methodology optimization. J. Chromatogr. A.

[47] Haghbakhsh R, Parvaneh K, Raeissi S, Shariati A (2018). A general viscosity model for deep eutectic solvents: The free volume theory coupled with association equations of state. Fluid Phase Equilib..

[48] Yong KJ, Wu TY (2022). Second-generation bioenergy from oilseed crop residues: Recent technologies, techno-economic assessments and policies. Energy Convers. Manag..

[49] Yue Y, Han J, Han G, Aita GM, Wu Q (2015). Cellulose fibers isolated from energycane bagasse using alkaline and sodium chlorite treatments: Structural, chemical and thermal properties. Ind. Crops Prod..

[50] Hajidariyor T (2023). Cryo-Induced cellulose-based nanogel from *Elaeis*
*guineensis* for antibiotic delivery platform. Int. J. Mol. Sci..

[51] Jonglertjunya W, Juntong T, Pakkang N, Srimarut N, Sakdaronnarong C (2014). Properties of lignin extracted from sugarcane bagasse and its efficacy in maintaining postharvest quality of limes during storage. LWT Food Sci. Technol..

[52] Goering HK (1970). Forage Fiber Analyses (Apparatus, Reagents, Procedures, and Some Applications).

[53] Pangsang N (2019). Chemical-free fractionation of palm empty fruit bunch and palm fiber by hot-compressed water technique for ethanol production. Energy Rep..

[54] Sakdaronnarong C (2018). Integrative process for a sugarcane bagasse biorefinery to produce glucose, bio-oil and carbon microspheres. Process Saf. Environ. Prot..

[55] Sakdaronnarong C, Jiratanakittiwat K, Tangkitthanasakul T, Laosiripojana N (2017). Ionosolv pretreatment of sugarcane bagasse and rice straw assisted by catalytic hydrothermal and microwave heating for biorefining. Food Bioprod. Process..

[56] Segal L, Creely JJ, Martin AE, Conrad CM (1959). An empirical method for estimating the degree of crystallinity of native cellulose using the X-ray diffractometer. Textile Res. J..

[57] Ma Y (2019). Production of nanocellulose using hydrated deep eutectic solvent combined with ultrasonic treatment. ACS Omega.

[58] Satlewal A, Agrawal R, Bhagia S, Sangoro J, Ragauskas AJ (2018). Natural deep eutectic solvents for lignocellulosic biomass pretreatment: Recent developments, challenges and novel opportunities. Biotechnol. Adv..

[59] Nosri, W. *et al.* Production of cellooligosaccharides from oil palm bunch in bio-based choline chloride deep eutectic solvents and MALDI-TOF MS analysis of COS mixture. *Biomass Bioenergy* **180**, 107005. 10.1016/j.biombioe.2023.107005 (2024).

[60] Kumar AK (2020). Techno-economic evaluation of a natural deep eutectic solvent-based biorefinery: Exploring different design scenarios. Biofuels Bioprod. Biorefin..

[61] Zang G, Shah A, Wan C (2020). Techno-economic analysis of an integrated biorefinery strategy based on one-pot biomass fractionation and furfural production. J. Clean. Prod..

[62] Hossain MA (2021). Effects of polyol-based deep eutectic solvents on the efficiency of rice straw enzymatic hydrolysis. Ind. Crops Prod..

[63] Lenihan P (2010). Dilute acid hydrolysis of lignocellulosic biomass. Chem. Eng. J..

[64] Bondancia TJ (2020). Production of nanocellulose using citric acid in a biorefinery concept: Effect of the hydrolysis reaction time and techno-economic analysis. Ind. Eng. Chem. Res..

[65] Guo J, Guo X, Wang S, Yin Y (2016). Effects of ultrasonic treatment during acid hydrolysis on the yield, particle size and structure of cellulose nanocrystals. Carbohydr. Polym..

[66] Wang J (2021). Preparation of nanocellulose in high yield via chemi-mechanical synergy. Carbohydr. Polym..

[67] Li Y (2016). Facile extraction of cellulose nanocrystals from wood using ethanol and peroxide solvothermal pretreatment followed by ultrasonic nanofibrillation. Green Chem..

[68] Hongrattanavichit I, Aht-Ong D (2020). Nanofibrillation and characterization of sugarcane bagasse agro-waste using water-based steam explosion and high-pressure homogenization. J. Clean. Prod..

[69] Gao C, Yang J, Zhang H, Xiao W, Han L (2020). Quantitative and qualitative characterization of dual scale mechanical enhancement on cellulosic and crystalline-structural variation of NaOH treated wheat straw. Bioresour. Technol..

[70] Li X, Tang W, He Y-C (2023). Integrated understanding of acidic deep eutectic solvent choline chloride: Oxalic acid pretreatment to enhance the enzymatic hydrolysis of rape straw. Ind. Crops Prod..

[71] Kong L, Fan B, He Y-C (2023). Efficient whole-cell biosynthesis of (S)-2-chloro-1-(3,4-difluorophenyl)-ethanol from 2-chloro-1-(3,4-difluorophenyl) ethanone in a sustainable reaction system. Mol. Catal..

[72] Wojeicchowski JP, Abranches DO, Ferreira AM, Mafra MR, Coutinho JAP (2021). Using COSMO-RS to predict solvatochromic parameters for deep eutectic solvents. ACS Sustain. Chem. Eng..

[73] Zhang Y (2023). Mechanistic insights into the lignin dissolution behavior in amino acid based deep eutectic solvents. Int. J. Biol. Macromol..

[74] Chen Y, Ma C, Tang W, He Y-C (2023). Comprehensive understanding of enzymatic saccharification of Betaine: Lactic acid-pretreated sugarcane bagasse. Bioresour. Technol..

[75] Jessop PG, Jessop DA, Fu D, Phan L (2012). Solvatochromic parameters for solvents of interest in green chemistry. Green Chem..

[76] Mulia K, Fauzia F, Krisanti EA (2019). Polyalcohols as hydrogen-bonding donors in choline chloride-based deep eutectic solvents for extraction of xanthones from the pericarp of *Garcinia*
*mangostana* L. Molecules.

[77] Harris RC (2009). Physical Properties of Alcohol Based Deep Eutectic Solvents.

[78] Thi S, Lee KM (2019). Comparison of deep eutectic solvents (DES) on pretreatment of oil palm empty fruit bunch (OPEFB): Cellulose digestibility, structural and morphology changes. Bioresour. Technol..

[79] Saini JK, Patel AK, Adsul M, Singhania RR (2016). Cellulase adsorption on lignin: A roadblock for economic hydrolysis of biomass. Renew. Energy.

[80] Li Z (2016). Effect of extractives on digestibility of cellulose in corn stover with liquid hot water pretreatment. BioResources.

[81] Zhang H (2020). Study on the dissolution mechanism of cellulose by ChCl-based deep eutectic solvents. Materials (Basel).

[82] Wang Y, Yang Y, Qu Y, Zhang J (2021). Selective removal of lignin with sodium chlorite to improve the quality and antioxidant activity of xylo-oligosaccharides from lignocellulosic biomass. Bioresour. Technol..

[83] Ceaser R, Rosa S, Montané D, Constantí M, Medina F (2023). Optimization of softwood pretreatment by microwave-assisted deep eutectic solvents at high solids loading. Bioresour. Technol..

[84] Nazir MS, Wahjoedi BA, Yussof AW, Abdullah MA (2013). Eco-friendly extraction and characterization of cellulose from oil palm empty fruit bunches. BioResources.

[85] Xia Q (2018). Multiple hydrogen bond coordination in three-constituent deep eutectic solvents enhances lignin fractionation from biomass. Green Chem..

[86] Yuan J-C (2023). Facile production of cellulose nanofibers from raw elephant grass by an aluminum chloride-enhanced acidic deep eutectic solvent. Int. J. Biol. Macromol..

[87] Ji Q, Yu X, Yagoub AE-GA, Chen L, Zhou C (2021). Efficient cleavage of strong hydrogen bonds in sugarcane bagasse by ternary acidic deep eutectic solvent and ultrasonication to facile fabrication of cellulose nanofibers. Cellulose.

[88] Guo Z, Ling Z, Wang C, Zhang X, Xu F (2018). Integration of facile deep eutectic solvents pretreatment for enhanced enzymatic hydrolysis and lignin valorization from industrial xylose residue. Bioresour. Technol..

[89] Nishiyama Y, Langan P, Chanzy H (2002). Crystal structure and hydrogen-bonding system in cellulose Iβ from synchrotron X-ray and neutron fiber diffraction. J. Am. Chem. Soc..

[90] Thulluri C, Balasubramaniam R, Velankar HR (2021). Generation of highly amenable cellulose-Iβ via selective delignification of rice straw using a reusable cyclic ether-assisted deep eutectic solvent system. Sci. Rep..

[91] Thomas LH (2015). Diffraction evidence for the structure of cellulose microfibrils in bamboo, a model for grass and cereal celluloses. BMC Plant Biol..

[92] Nakagaito AN, Yano H (2005). Novel high-strength biocomposites based on microfibrillated cellulose having nano-order-unit web-like network structure. Appl. Phys. A.

[93] Davoudpour Y (2015). Optimization of high pressure homogenization parameters for the isolation of cellulosic nanofibers using response surface methodology. Ind. Crops Prod..

[94] Chaker A, Alila S, Mutjé P, Vilar MR, Boufi S (2013). Key role of the hemicellulose content and the cell morphology on the nanofibrillation effectiveness of cellulose pulps. Cellulose.

[95] Jonoobi M, Harun J, Mathew AP, Hussein MZB, Oksman K (2010). Preparation of cellulose nanofibers with hydrophobic surface characteristics. Cellulose.

