# Supplementary material for: Process design for acidic and alcohol based deep eutectic solvent pretreatment and high pressure homogenization of palm bunches for nanocellulose production
Source: Sci Rep. 2024 Mar 30;14:7550. doi: 10.1038/s41598-024-57631-9 (PMC10981746; doi:10.1038/s41598-024-57631-9)
Supplement: Supplementary file 1 — Supplementary Information. [file 41598_2024_57631_MOESM1_ESM.docx]

**Supporting Information**

**Process Design for Acidic and Alcohol Based Deep Eutectic Solvent Pretreatment and High Pressure Homogenization of Palm Bunches for Nanocellulose Production**

Janejira Sonyeam^a,✝^, Ratanaporn Chaipanya^a,✝^, Sudarat Suksomboon^a,✝^, Mohd Jahir Khan^a,✝^, Krongkarn Amatariyakul^a^, Agung Wibowo^a^, Pattaraporn Posoknistakul^a^, Boonya Charnnok^b^, Chen Guang Liu^c^, Navadol Laosiripojana^d^, Chularat Sakdaronnarong^a^,*

^a^ Department of Chemical Engineering, Faculty of Engineering, Mahidol University, 25/25

Putthamonthon 4 Road,Salaya, Putthamonthon, Nakhon Pathom 73170 Thailand

^b^ Department of Specialized Engineering, Energy Technology Program, Faculty of Engineering, Prince of Songkla University, Hat Yai District, Songkhla Province, 90110, Thailand

^c^ State Key Laboratory of Microbial Metabolism, School of Life Sciences and Biotechnology, Shanghai Jiao Tong University, 800 Dongchuan Road, Shanghai 200240, China

^d^ The Joint Graduate School of Energy and Environment, King Mongkut's University of Technology Thonburi, 126 Pracha Uthit Road, Bang Mot, Thung Khru, Bangkok 10140 Thailand

^✝^ The authors contributed equally as co-first authors

* Corresponding author’s email address: Chularat.sak@mahidol.ac.th

**Table S1.** Mass balance of simulated process of cellulose extraction from EFB in ChCl/1,3-butanediol (ChBu) at 200 °C for 20 min (CellSep-A1 process)

| Line No | 1 | 2 | 3 | 4 | 5 | 6 | 7 |
| --- | --- | --- | --- | --- | --- | --- | --- |
| Stream component | Material Feed | Sulfuric acid feed | Acid Hydrolysis output | Water feed | Mix tank output | Centrifuge output | CNC |
| Cellulose (kg/h) | 74 |  |  |  |  |  |  |
| Hemicellulose (kg/h) | 19 |  |  |  |  |  |  |
| Lignin (kg/h) | 6 |  | 6 |  | 6 | 6 |  |
| Others (kg/h) | 1 |  | 23 |  | 23 | 23 |  |
| Sulfuric acid (kg/h) |  | 1765.44 | 1765.44 |  | 1765.44 | 1765.44 |  |
| Water (kg/h) |  | 993.1 | 993.1 | 15000 | 15993.1 | 15993.1 |  |
| CNC (kg/h) |  |  | 71 |  | 71 |  | 71 |
| Total | 100 | 2758.5 | 2858.5 | 15000 | 17858.5 | 177788 | 71 |
| Pressure (bar) | 1 | 1 | 1 | 1 | 1 | 1 | 1 |
| Temperature (°C) | 25 | 25 | 45 | 25 | 25 | 25 | 25 |

**Table S2.** Cost estimate of the studies on cellulose separation (CellSep process)

|  | **CellSep-A1** | **CellSep-A2** | **CellSep-A3** |
| --- | --- | --- | --- |
|  | **Nosri et al. 2023**^1^ | **Kumar et al. 2020**^2^ | **Zang et al. 2020**^3^ |
| **1.Fixed capital cost (USD)** | 1,161,671.58 | 1,262,462.39 | 2,215,988.66 |
| **2.Total operating cost (USD/yr)** | 33,137,826.46 | 23,127,103.01 | 22,949,146.20 |
| **- Operating labour cost (USD/yr)** | 77,215.69 | 77,215.69 | 186,950.71 |
| **- Utility cost (USD/yr)** | 5,023,695.56 | 4,991,381.84 | 11,300,832.29 |
| **- Raw material cost (USD/yr)** | 28,036,915.20 | 18,058,505.47 | 11,461,363.20 |
| **3. Revenue (USD/yr)** | 33,699,859.20 | 23,335,603.20 | 23,228,755.20 |
| **Ratio Revenue/operating cost** | 1.02 | 0.96 | 1.01 |
| **Discount rate** | 0.08 | 0.08 | 0.08 |
| **Present value of future cash flows (USD)** | 2,416,309.13 | 963,871.30 | 1,455,747.92 |
| **Initial Investment (USD)** | 1,161,671.58 | 1,262,462.39 | 2,215,988.66 |
| **NPV (USD)** | 1,254,637.55 | -298,591.09 | -760,240.74 |
| **IRR** | 47% | 0% | -14% |
| **Pay Back (year)** | 3 | 7 | 8 |

**Table S3.** Mass balance of simulated process of CNC production from softwood sulfite pulp using 64 wt% sulfuric acid at 45 °C for 2 h ^4^ (ChemCNC-B2 process)

| Line No | 1 | 2 | 3 | 4 | 5 | 6 | 7 |
| --- | --- | --- | --- | --- | --- | --- | --- |
| Strem component | Material Feed | Sulfuric acid feed | Acid Hydrolysis output | Water feed | Mix tank output | Centrifuge output | CNC |
| Cellulose (kg/h) | 74 | - | - | - | - | - | - |
| Hemicellulose (kg/h) | 19 | - | - | - | - | - | - |
| Lignin (kg/h) | 6 | - | 6 | - | 6 | 6 | - |
| Others (kg/h) | 1 | - | 23 | - | 23 | 23 | - |
| Sulfuric acid (kg/h) | - | 1765.44 | 1765.44 | - | 1765.44 | 1765.44 | - |
| Water (kg/h) | - | 993.06 | 993.06 | 15000 | 15993.1 | 15993.1 | - |
| CNC (kg/h) | - | - | 71 | - | 71 | - | 71 |
| Total | 100 | 2758.5 | 2858.5 | 15000 | 17858.5 | 177788 | 71 |
| Pressure (bar) | 1 | 1 | 1 | 1 | 1 | 1 | 1 |
| Temperature (°C) | 25 | 25 | 45 | 25 | 25 | 25 | 25 |

**Table S4.** Cost estimate of the studies on chemical process for nanocellulose production (ChemCNC process)

|  | **ChemCNC-B1** | **ChemCNC-B2** | **ChemCNC-B3** |
| --- | --- | --- | --- |
|  | **Bondancia et al. 2020** ^5^ | **Guo et al. 2015** ^4^ | **Wang et al. 2020** ^6^ |
| **1.Fixed capital cost (USD)** | 12,521,855.48 | 3,021,729.26 | 964,704,70 |
| **2.Total operating cost (USD/yr)** | 281,461,006,52 | 866,339,712.81 | 192,542,141.90 |
| **- Operating labour cost (USD/yr)** | 81,087.51 | 77,109.90 | 77,109.90 |
| **- Utility cost (USD/yr)** | 276,097,942.83 | 184,291,634.91 | 189,571,448.30 |
| **- Raw material cost (USD/yr)** | 5,281,976.18 | 681,970,968 | 2,893,583.70 |
| **3. Revenue (USD/yr)** | 281,912,400 | 866,484,000 | 192,579,120 |
| **Ratio Revenue/operating cost** | 1.00 | 1.00 | 1.00 |
| **Discount rate** | 0.08 | 0.08 | 0.08 |
| **Present value of future cash flows (USD)** | 5,081,690.02 | 1,624,353.92 | 416,291.43 |
| **Initial Investment (USD)** | 12,521,855.48 | 3,021729.26 | 964,704,70 |
| **NPV (USD)** | -7,440,165.47 | -1,397,375.35 | -548,413.27 |
| **IRR** | 1% | 2% | 1% |
| **Pay Back (year)** | 28 | 21 | 27 |

**Table S5.** Mass balance of simulated process of microcrystalline production from pretreated wheat straw using a combined ball milling and alkaline treatment^7^ (MechCN-C3 process)

|  | 1 | 2 | 3 | 4 | 5 | 6 | 7 | 8 |
| --- | --- | --- | --- | --- | --- | --- | --- | --- |
| Number of Stream | EFB | Choline Chloride | 1,3-Butanediol | DESs | Slurry A | Water | Solid A | Slurry B |
| Cellulose (kg/h) | 38.5 |  |  |  | 38.5 |  | 17.6 | 20.9 |
| Hemicellulose (kg/h) | 26.1 |  |  |  | 26.1 |  |  | 26.1 |
| Lignin (kg/h) | 18.9 |  |  |  | 18.9 |  |  | 18.9 |
| Others (kg/h) | 16.5 |  |  |  | 16.5 |  |  | 37.4 |
| Choline Chloride (kg/h) |  | 500 |  | 435.2 | 435.2 |  |  | 435.2 |
| 1,3-Butanediol (kg/h) |  |  | 649 | 564.8 | 564.8 |  |  | 564.8 |
| Ethanol (kg/h) |  |  |  |  |  |  |  |  |
| Water (kg/h) |  |  |  |  |  | 2534.67 |  | 2534.67 |
| Total | 100 | 500 | 649 | 1000 | 1100 | 2534.67 | 17.6 | 3637.97 |
| Pressure (bar) | 1 | 1 | 1 | 1 | 20 | 1 | 1 | 1 |
| Temperature (°C) | 30 | 30 | 30 | 30 | 200 | 30 | 30 | 30 |

**Table S6.** Cost estimate of the studies on nanocellulose production (mechanical process)

|  | **MechCN-C1** | **MechCN-C2** | **MechCN-C3** |
| --- | --- | --- | --- |
|  | **Li et al. 2016** ^8^ | **Hongrattanavichit et al. 2020** ^9^ | **Gao et al. 2020** ^7^ |
| **1.Fixed capital cost (USD)** | 10,091,383.23 | 10,981,796.08 | 1,570,885.45 |
| **2.Total operating cost (USD/yr)** | 40,039,720.73 | 43,473,648.35 | 14,120,534.40 |
| **- Operating labour cost (USD/yr)** | 79,783.67 | 82,370.71 | 77,109.90 |
| **- Utility cost (USD/yr)** | 39,094,443.57 | 43,237,734.56 | 13,972,551.83 |
| **- Raw material cost (USD/yr)** | 865,493.48 | 153,543.08 | 70,872.68 |
| **3. Revenue (USD/yr)** | 46,559,520.00 | 44,974,800 | 15,467,760 |
| **Ratio Revenue/operating cost** | 1.16 | 1.03 | 1.10 |
| **Discount rate** | 0.08 | 0.08 | 0.08 |
| **Present value of future cash flows (USD)** | 26,031,668.00 | 12,849,075.57 | 2,402,459.91 |
| **Initial Investment (USD)** | 10,091,383.23 | 10,981,796.08 | 1,570,885.45 |
| **NPV (USD)** | 15,940,284.77 | 1,867,279.48 | 831,574.46 |
| **IRR** | 58% | 11% | 45% |
| **Pay Back (year)** | 2 | 8 | 3 |

**Table S7.** Economic analysis of DES cost based on 10g EFB: 100g DES pretreatment of the best condition providing highest cellulose yield from ChOx, ChBu and ChLa pretreatment.

| HBA and HBD | HBA:HBD (mol:mol) | HBA:HBD based on MW (g:g) | HBA:HBD weight ratio in total 1 g | Weight of chemicals for 100g DES (g) | Chemical price (Alfa Aesar 2024)^B^ | Chemical price (USD/kg or USD/L) | Chemical cost of DES for 10g EFB (USD) | %Cellulose after bleaching based on raw EFB^A^ | Bleached cellulose produced from 10 g raw EFB (g) | DES cost (USD/g cellulose) |
| --- | --- | --- | --- | --- | --- | --- | --- | --- | --- | --- |
| ChCl (MW 139.62) | 1 | 139.62 | 0.61 | 60.80 | 446 USD/10 kg | 44.60 | 2.71 | 6.80 | 0.26 | 30.57 |
| Oxalic acid (MW 90.03) | 1 | 90.03 | 0.39 | 39.20 | 135 USD/1kg | 135.00 | 5.29 |  |  |  |
|  |  |  |  |  |  | Total cost | 8.00 | ChOx80C |  |  |
| ChCl (MW 139.62) | 1 | 139.62 | 0.44 | 43.65 | 446 USD/10 kg | 44.60 | 1.95 | 50.30 | 1.94 | 1.75 |
| 1,3-Butanediol (MW 90.12) | 2 | 180.24 | 0.56 | 56.35 | 255USD/10L | 25.50 | 1.44 |  |  |  |
|  |  |  |  |  |  | Total cost | 3.38 | ChBu60C |  |  |
| ChCl (MW 139.62) | 1 | 139.62 | 0.13 | 13.42 | 446 USD/10 kg | 44.60 | 0.60 | 68.50 | 2.64 | 2.09 |
| Lactic acid (MW 90.08) | 10 | 900.8 | 0.87 | 86.58 | 142 USD/2.5kg | 56.80 | 4.92 |  |  |  |
|  |  |  |  |  |  | Total cost | 5.52 | ChLa80C |  |  |

Note The calculation was based on DES pretreatment of 10g EFB : 100 g DES

^A^ is %cellulose yield after bleaching based on cellulose content of raw EFB as 38.5% from different DES treatment namely ChOx80C, ChBu60C and ChLa80C according to the data from Table 1.

^B^ Price of chemicals was calculated based on Alfa Aesar supplier: choline chloride (>98%, J13410), oxalic acid (>99.5% ACS, 033262), 1,3-butanediol (~99%, A11684), and DL-lactic acid (80-85%, L14259)

**Table S8.** The summary of L/d ratios, morphology and viscosity tested at the sweeping frequency of 0.1 Hz of all nanocellulose samples

| Samples | 12-pass HPH  nanocellulose morphology | L/d ratio | Viscosity of ultrasonicated nanocellulose (Pa⋅s) | Viscosity of 12-pass HPH nanocellulose (Pa⋅s) |
| --- | --- | --- | --- | --- |
| ChLa60C  ChOx80C ChOx100C | Nanocrystals | 5-10 | 2.825  3.385  4.388 | 99.42  32.54  41.10 |
| ChBu80C | Nanofibers | ~65.8 | <1.0 | 12.92 |
| ChLa100C |  | ~17.4 to ~34.7 | 2.021 | 14.4 |
| ChBu60C  ChLa80C | Clumpy structure with lots of fiber aggregation | >100 | 4.898  13.92 | 147.90  46.65 |

Note: Viscosity of nanocellulose was measured using sweep test method at the frequency of 0.1 Hz.


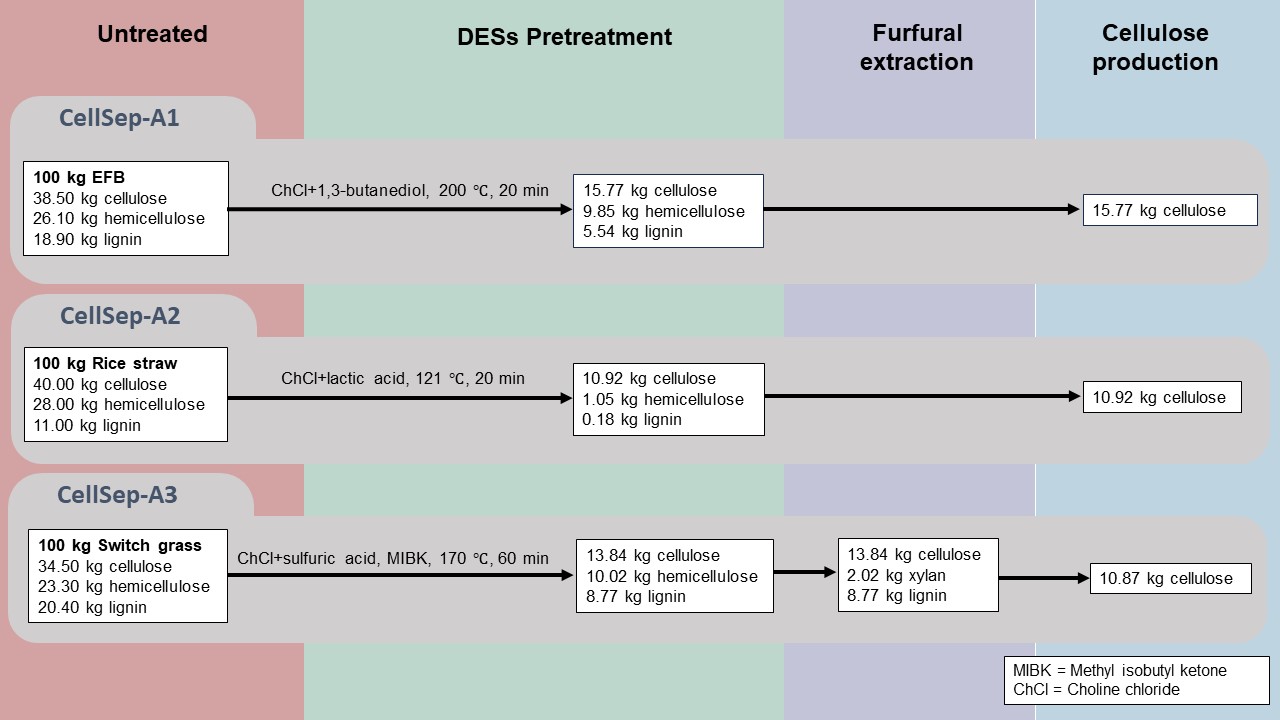


**Fig. S1** Comparison of mass balance of cellulose separation process (CellSep-A1, A2 and A3 processes) from lignocellulosic biomass based on previous researches.


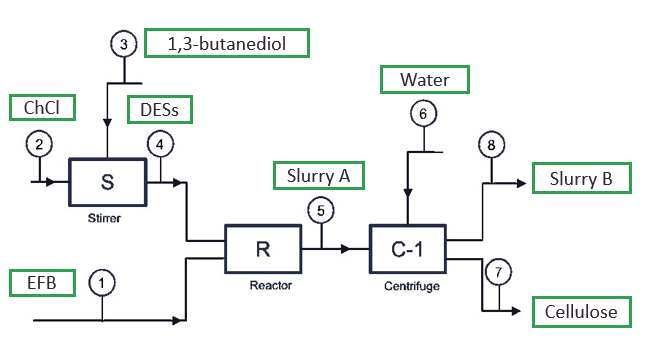


**Fig. S2** The optimal cellulose extraction process from EFB in Choline chloride (ChCl)/1,3-butanediol (ChBu) at 200 °C for 20 min (CellSep-A1 process)

**
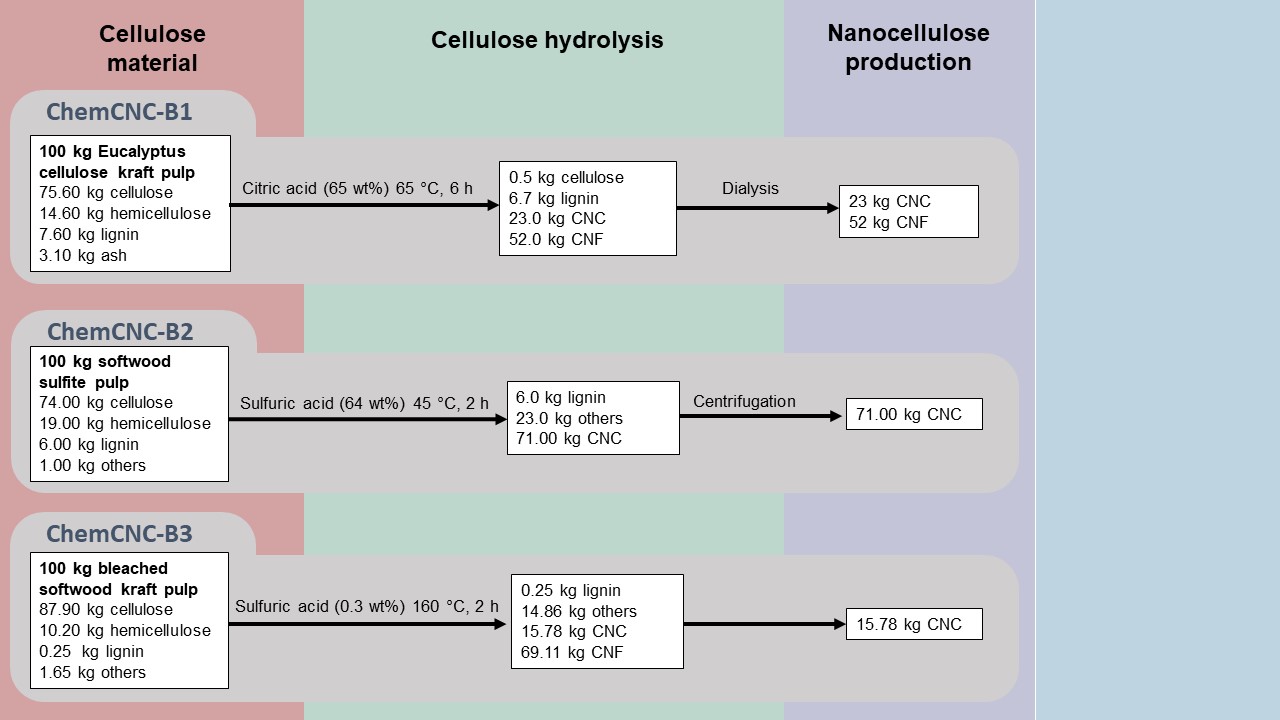
**

**Fig. S3** Mass balance of nanocellulose production from cellulosic pulp through chemical process (CellCNC-B1, B2 and B3 processes) based on previous researches


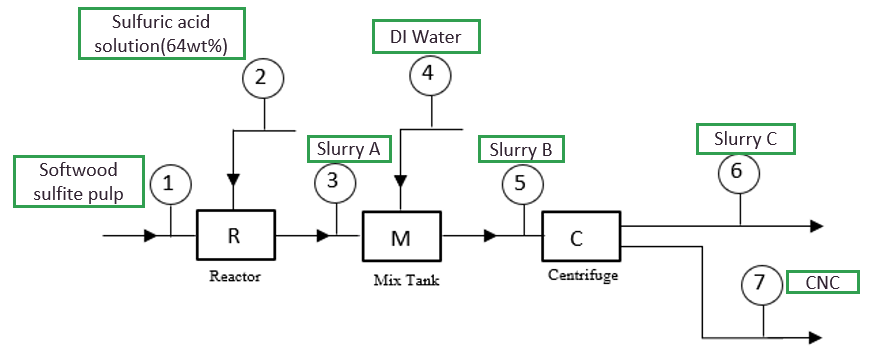


**Fig. S4** The optimal CNC production from cellulosic softwood sulfite pulp using 64 wt% sulfuric acid at 45 °C for 2 h (ChemCNC-B2 process)^4^.


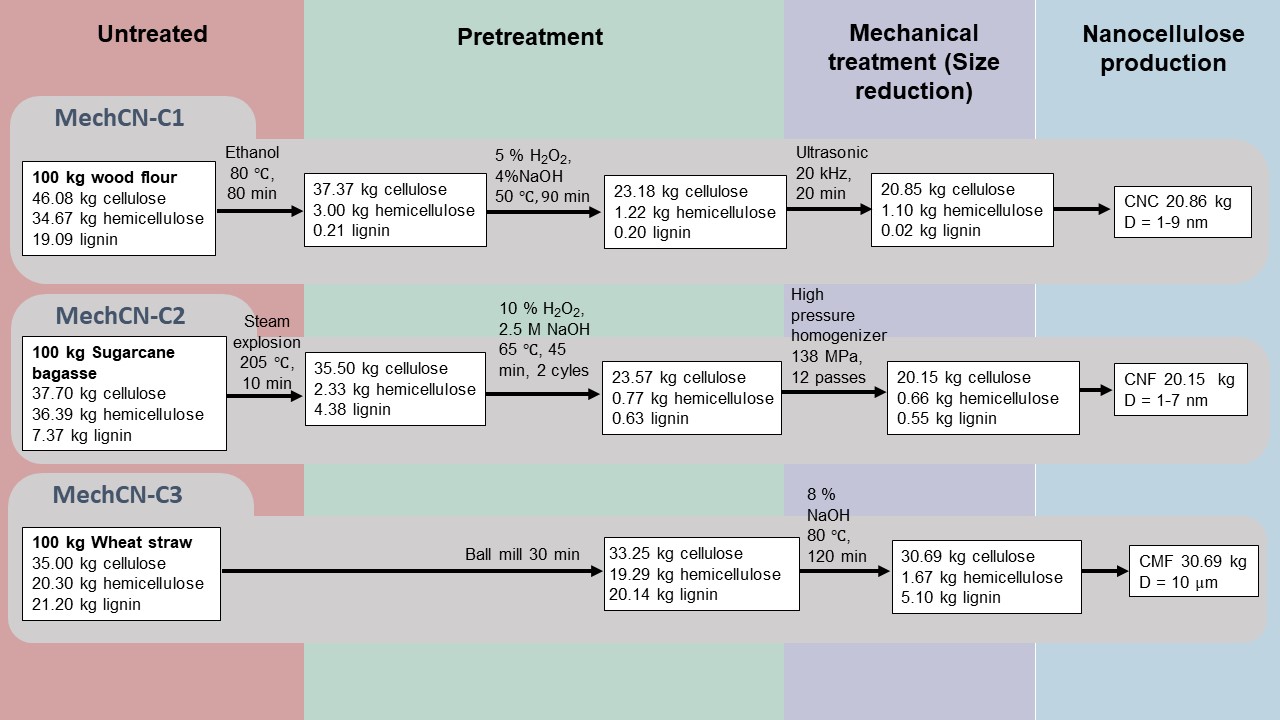


**Fig. S5** Mass balance of nanocellulose production from lignocellulosic biomass using mechanical process (MechCN-C1, C2 and C3 processes) based on previous researches


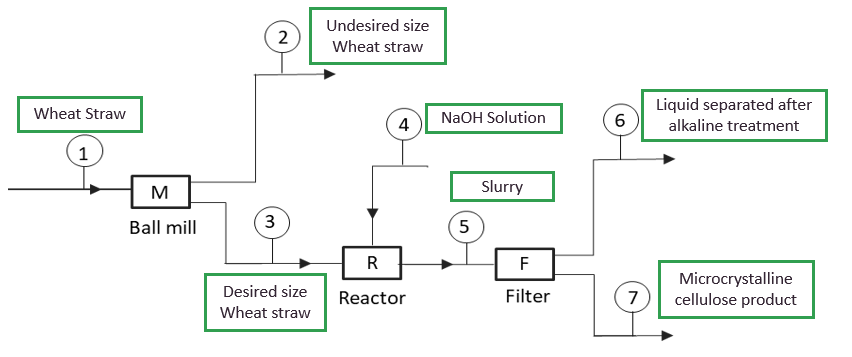


**Fig. S6** Optimal mechanical size reduction for microcrystalline production from pretreated wheat straw using a combined ball milling and alkaline treatment^7^ (MechCN-C3 process)

**Fig. S7** HPLC analysis of liquid DES after EFB pretreatment in different DES at 60 °C, 80 °C and 100 °C for 8 h.


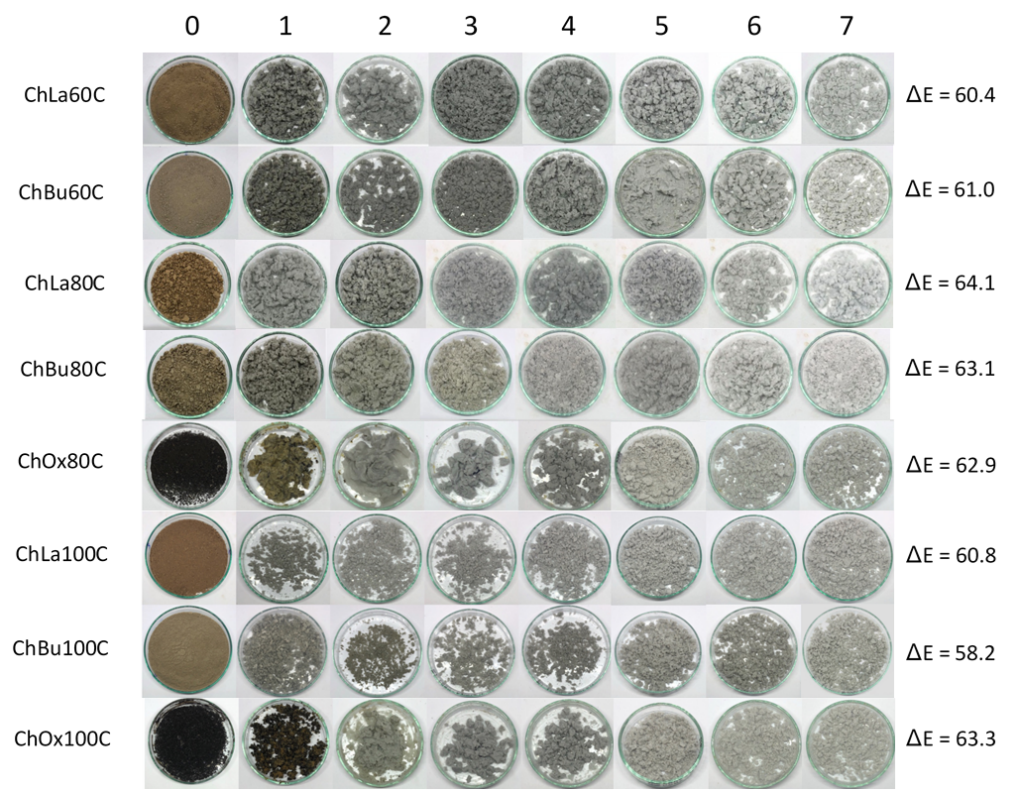


**Fig. S8** Color change and whiteness (ΔE) of bleached cellulose from different DES pretreatment methods from 1^st^ cycle to 7^th^ cycle of bleaching process.

**Fig. S9** Crystallinity index (CrI) of nanocellulose samples after ultrasonication and HPH for 0 to12 passes at 150 MPa of different DES pretreated and bleached cellulose namely ChLa60C, ChBu60C, ChLa80C, ChBu80C, ChOx80C, ChLa100C, ChBu100C, and ChOx100.

**References**

1 Nosri, W. *et al.* Conversion of cellulose extracted from oil palm empty fruit bunch to functional cellooligosaccharides in choline chloride based deep eutectic solvents. *Biomass and Bioenergy* **(under review)** (2023).

2 Kumar, A. K. *et al.* Techno-economic evaluation of a natural deep eutectic solvent-based biorefinery: Exploring different design scenarios. *Biofuels, Bioproducts and Biorefining* **14**, 746-763, doi:https://doi.org/10.1002/bbb.2110 (2020).

3 Zang, G., Shah, A. & Wan, C. Techno-economic analysis of an integrated biorefinery strategy based on one-pot biomass fractionation and furfural production. *J. Cleaner Prod.* **260**, 120837, doi:https://doi.org/10.1016/j.jclepro.2020.120837 (2020).

4 Guo, J., Guo, X., Wang, S. & Yin, Y. Effects of ultrasonic treatment during acid hydrolysis on the yield, particle size and structure of cellulose nanocrystals. *Carbohydr. Polym.* **135**, 248-255, doi:https://doi.org/10.1016/j.carbpol.2015.08.068 (2016).

5 Bondancia, T. J. *et al.* Production of Nanocellulose Using Citric Acid in a Biorefinery Concept: Effect of the Hydrolysis Reaction Time and Techno-Economic Analysis. *Ind. Eng. Chem. Res.* **59**, 11505-11516, doi:10.1021/acs.iecr.0c01359 (2020).

6 Wang, J. *et al.* Preparation of nanocellulose in high yield via chemi-mechanical synergy. *Carbohydr. Polym.* **251**, 117094, doi:https://doi.org/10.1016/j.carbpol.2020.117094 (2021).

7 Gao, C., Yang, J., Zhang, H., Xiao, W. & Han, L. Quantitative and qualitative characterization of dual scale mechanical enhancement on cellulosic and crystalline-structural variation of NaOH treated wheat straw. *Bioresour. Technol.* **312**, 123535, doi:https://doi.org/10.1016/j.biortech.2020.123535 (2020).

8 Li, Y. *et al.* Facile extraction of cellulose nanocrystals from wood using ethanol and peroxide solvothermal pretreatment followed by ultrasonic nanofibrillation. *Green Chem.* **18**, 1010-1018, doi:10.1039/C5GC02576A (2016).

9 Hongrattanavichit, I. & Aht-Ong, D. Nanofibrillation and characterization of sugarcane bagasse agro-waste using water-based steam explosion and high-pressure homogenization. *J. Cleaner Prod.* **277**, 123471, doi:https://doi.org/10.1016/j.jclepro.2020.123471 (2020).
